# Monolayer Stress Microscopy: Limitations, Artifacts, and Accuracy of Recovered Intercellular Stresses

**DOI:** 10.1371/journal.pone.0055172

**Published:** 2013-02-28

**Authors:** Dhananjay T. Tambe, Ugo Croutelle, Xavier Trepat, Chan Young Park, Jae Hun Kim, Emil Millet, James P. Butler, Jeffrey J. Fredberg

**Affiliations:** 1 Department of Environmental Health, Harvard School of Public Health, Boston, Massachusetts, United States of America; 2 Department of Physics, University Paris VII Diderot, Paris, France; 3 Institute for Bioengineering of Catalonia - ICREA, Barcelona, Spain; 4 Unitat de Biofísica i Bioenginyeria - CIBERES, Universitat de Barcelona, Barcelona, Spain; 5 Department of Medicine, Harvard Medical School, Boston, Massachusetts, United States of America; University of California, Berkeley, United States of America

## Abstract

In wound healing, tissue growth, and certain cancers, the epithelial or the endothelial monolayer sheet expands. Within the expanding monolayer sheet, migration of the individual cell is strongly guided by physical forces imposed by adjacent cells. This process is called plithotaxis and was discovered using Monolayer Stress Microscopy (MSM). MSM rests upon certain simplifying assumptions, however, concerning boundary conditions, cell material properties and system dimensionality. To assess the validity of these assumptions and to quantify associated errors, here we report new analytical, numerical, and experimental investigations. For several commonly used experimental monolayer systems, the simplifying assumptions used previously lead to errors that are shown to be quite small. Out-of-plane components of displacement and traction fields can be safely neglected, and characteristic features of intercellular stresses that underlie plithotaxis remain largely unaffected. Taken together, these findings validate Monolayer Stress Microscopy within broad but well-defined limits of applicability.

## Introduction

The human body is a cooperative of about 80 trillion cells, each one of which receives constantly from its neighbors both chemical and physical signals [1]. The individual cell then integrates these signals and responds through apoptosis, proliferation, differentiation, or migration. For example, pivotal events in the biology of the stem cell, the differentiated cell, and the cancer cell are increasingly understood to be of a mechanical nature [2]–[15]. These basic processes, in turn, underlie collective cellular processes that include morphogenesis, injury and repair, growth, regeneration, and cancer.

In collective migratory processes, cells ordinarily move not as individual entities but as collective sheets, ducts, strands, or clusters [8]. It is well established that each individual cell can follow preset chemical, adhesive, or mechanical gradients (chemotaxis, haptotaxis, and durotaxis, respectively [16]–[18]), but how each cell can coordinate its migration with that of immediate neighbors has defied full comprehension. For the cell-cell pair that is studied in isolation, mechanical stress exerted across the cell-cell junction has been recently quantified and is conceptually straightforward [19], [20]. For the integrated multicellular monolayer sheet, by contrast, mechanical stresses exerted across the multiple cell-cell junctions amongst numerous immediate neighbors have been more difficult to quantify experimentally.

For the particular case of the cellular monolayer *in vitro*, the stresses exerted between a cell and its neighbors can now be mapped using the method called Monolayer Stress Microscopy (MSM) [21], [22]. Using MSM, Tambe et al. [22] have recently shown that the stresses within and between cells comprising an advancing monolayer sheet define a remarkably rugged stress landscape. And within that landscape, the individual cell tends to migrate along the local orientation of maximal principal stress in a collective migratory process referred to as plithotaxis [21]–[25]. Collective cellular migration, a heterogeneous stress landscape, and plithotaxis all arise in the context of multicellular cooperative systems but logically cannot arise in single cells or in isolated cell-cell pair interactions.

Implementation of MSM begins with recovery of local tractions exerted by the monolayer upon its substrate. As originally described [26], [27], the traction recovery procedure is two-dimensional in the sense that components of both the local traction field and the local displacement field that are out of the plane of the monolayer are assumed to be negligible. Others have subsequently disputed the validity of this assumption on the grounds that out-of-plane tractions and out-of-plane displacements may be appreciable [28], [29]. For estimation of in-plane tractions, they have argued that three dimensional methods are necessary because out-of-plane events invalidate two-dimensional methods [28], [29]. We disagree with this argument, and present in Results and Discussion detailed supporting evidence.

Whether or not two-dimensional conditions might obtain, still other assumptions are required. The distribution of internal stresses within the monolayer is computed from the recovered in-plane tractions based on the two-dimensional balance of forces in the cell plane as demanded by Newton's laws [22]. As described below, this balance of forces is expressed as a two-dimensional elasto-static boundary value problem (BVP) with a source term corresponding to the local substrate traction. To solve this problem requires two key assumptions. The first involves the physical conditions defined by stresses, displacements, or some combination thereof at the monolayer boundary. The optical field-of-view is sometimes limited to a small region of the monolayer, and the boundary conditions at this limit are unknown. The second involves the elastic properties of the monolayer itself. The monolayer elastic properties are unknown as well. Concerning these unknowns, Tambe et al. [22] made certain assumptions and performed limited analysis supporting their plausibility.

Here we reexamine these assumptions quantitatively in detail. We begin by formulating the problem in two dimensions and highlighting the necessary assumptions. We go on to consider boundary conditions and associated ambiguities, and then describe our analytical, numerical, and experimental approaches. Taken together, these approaches show quantitatively that errors attributable to out-of-plane tractions and displacements, and to cell material properties, are slight, and that errors attributable to unknown boundary conditions, if suitably handled, are manageable.

### Governing equations

**Figure 1:**
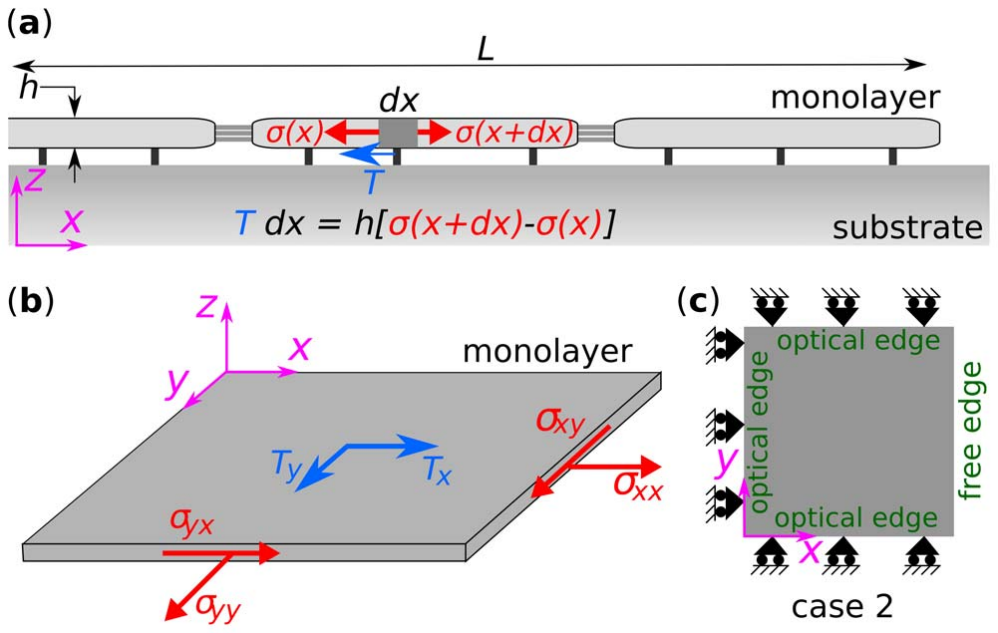
Balance of forces considered in MSM. (**a**) Cell monolayer is considered as a thin sheet of cells. Each cell in the monolayer exerts traction, 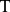, on the substrate. According to the Newton's second law, the tractions are balanced by local monolayer stress, 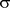, such that, in the one dimensional force balance, 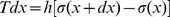. (**b**) The force balance is ensured only in the 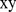 plane. Variation of stresses across the thickness is assumed to be negligible. (**c**) In classical wound healing assay, also referred to as case 2, the optical field-of-view has three optical edges and a free edge. For boundary conditions, all edges have shear stress to be zero. In addition, the free edge has normal stress to be zero and the optical edge has normal displacement to be zero.

We consider a monolayer comprising a collection of contiguous cells which forms a sheet that is flat and thin (Fig. 1a). By flat and thin we mean that, compared with the lateral span (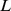) of the monolayer, its radius of out-of-plane curvature is large and its height (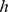) is small. In that case, stresses within the monolayer (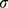) and underlying tractions exerted by the monolayer upon its substrate (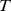) are taken to be planar with no out-of-plane contributions (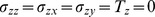, Fig. 1b); the contribution of out-of-plane tractions, and the accuracy of in-plane traction recovery is assessed in Results and Discussion.

If inertial effects are negligible, then according to Newton's second law, the local traction must be balanced by local gradients in monolayer stresses. This is simply a force balance argument, which implies
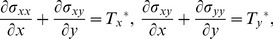
(1)where 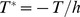. If the monolayer is homogeneous and isotropic, then there are only two independent elastic constants, which can be described by the Young's modulus 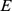 and the Poisson's ratio 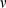; we consider the viscous properties in Discussion. In this case, the relationship between strain and stress is given by
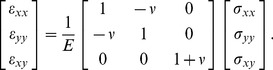
(2)To ensure that displacements can be integrated from strains yielding a single valued vector field, Saint-Venant's compatibility relation for strains must be satisfied, 
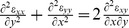
. Together with Eqs. 1 and 2, we then obtain the Beltrami-Michell compatibility equation,
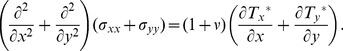
(3)
Eqs. 1 and 3 are sufficient to ensure local force balance; the source terms arising from tractions and the boundary conditions then determine stresses everywhere within the monolayer.

Since the monolayer always remains in mechanical equilibrium, Newton's laws demand that this force balance must be independent of material properties of the monolayer. The recovered stresses, however, are not independent of the specific material properties. Tambe et al. [22] had asserted incorrectly that the specific material properties of the monolayer have no effect on the recovered distribution of intercellular stresses; while this statement is indeed true for one-dimensional systems, it need not be true in two-dimensional systems largely due to effects of Poisson's ratio. Eqs. 1 and 3 show that if the material properties are homogeneous, then the distribution of internal stresses does not depend upon 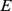 but does depend upon 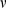. However, we show below that variations in 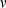 in two dimensions can have at most only a small effect. Thus, the prior assertion of Tambe et al. [22] that recovered stresses are independent of specific material properties remains true in one dimension and is approximately true in two dimensions.

In MSM, stresses are determined with standard finite element procedures which use Eqs. 1 and 2. In this case, Eq. 3 is satisfied automatically. Below we follow the standard approach and to do so we assign values for 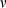 and 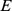. In Results we assess the influence of 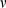 on the recovered monolayer stresses. In contrast, the assigned value for 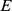 is entirely arbitrary, and does not influence the recovered monolayer stress distribution.

If the monolayer is neither isotropic, homogeneous, nor elastic, then the Beltrami-Michell compatibility equation becomes more complex, In Results and Discussion we deal with these cases as well.

### Boundary conditions

In most experimental monolayer systems, two types of boundaries require consideration (Fig. 1c). The first is the edge of the advancing monolayer front that is bounded by a cell-free, traction-free region; we call this the free edge. The second is the edge of the optical field-of-view that is bounded on one side by cells that are outside the optical field-of-view, and on the other side by cells that are inside the optical field-of-view; we call this the optical edge. Boundary conditions at the free edge are known (monolayer boundary stresses are zero), but boundary conditions at the optical edge are unknown.

To overcome the difficulty posed by the boundary conditions at the optical edge, Tambe et al. [22] assumed that along the optical edges 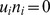, 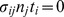, (summation convention used) where 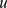 is the displacement, 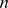 and 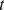 are the normal and tangent unit vectors at the optical edge; we call this a symmetric boundary condition. These boundary conditions imply that the region of interest (Fig. 1c) is a repeatable unit of the monolayer; the free edge defines the boundary of the monolayer, but the optical edge defines the interior edge across which the unit repeats as a mirror image. As such, Fig. 1c represents a monolayer that is infinite in extent along the 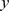-direction and twice the size of the region of interest along the 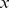-direction.

Recognizing that this assumption is *ad hoc* and might engender errors, Tambe et al. [22] showed by experimental means that these errors are largest in regions nearest the optical edge and decay rapidly with distance from the optical edge, becoming negligible beyond a distance of 20% of the length of the boundary. In this connection, the subsequent work of Hur et al. [28] assumes optical edges to be stress free (both normal and tangential stresses are zero); in Discussion, we provide below a quantitative comparison of the approach of Hur et al. [28] with that of Tambe et al. [22].

At the optical edge, Tambe et al. [22] constrained the normal displacements to be zero (Fig. 1c). In this case, a nonzero stress along the 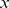-direction will induce stress along the 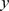-direction which is partly artifactual. Although this induced stress along the 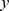-direction is associated with the boundary conditions, its magnitude depends upon 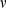. Therefore, in the approach of Tambe et al. [22], boundary artifacts and material artifacts are coupled.

In the measurement of monolayers bounded by free edges alone (Fig. S2c in File S1), there are no optical edges, and as such, there are no unknown boundary conditions and no associated boundary artifacts. We take advantage of this fact below.

## Analysis

Based on geometric scaling arguments, we first estimate the magnitude of out-of-plane tractions in comparison with in-plane tractions; based on analytic arguments, we then quantify the errors in recovered in-plane tractions when out-of-plane displacements are neglected. This is followed by an analysis of the errors in monolayer stresses attributable to the assumed simplified material properties of the cells. We conclude by considering the distinction between monolayers whose entire extent falls within the microscopic field-of-view versus those that extend outside the field-of-view.

### Analytic assessments

#### Out-of-plane tractions and displacements

Concerning traction recovery, we first consider the role of out-of-plane tractions, 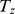 [27]. Based purely on geometric arguments, we begin with a preliminary estimation of the error. For a semi-infinite elastic medium, we then derive the full analytic Fourier representation of substrate tractions and displacements in three dimensions.

### Experimental assessments

To assess the accuracy of recovered monolayer stresses experimentally, we start by considering the monolayer bounded by free edges alone; we call such a monolayer a cell island (Fig. S2c, for experimental protocol see Supporting Information S1, in File S1). Stresses computed over the entire cell island have two useful properties. As noted above, the first property is the absence of optical edges and resulting boundary artifacts; accordingly we take these recovered stresses as being a gold standard. By comparison, we can then assess boundary artifacts in subsystems that include optical edges. The second property is that the absence of boundary artifacts makes these stresses ideal to assess MSM's artifacts that might be attributable to the assumptions of material incompressibility and homogeneity.

In this analysis, we compute local principal stress components (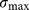 and 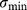), and focus on the local average normal stress 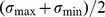 (which is the average local tension), the local maximum principal orientation (which is the axis of 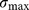 or local highest tension), and the local maximum shear stress 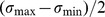 (which is a measure of the local stress anisotropy).

#### Effects of material properties of the monolayer

For 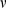, reported values range from 0.3 to 0.5 [30]. To assess the influence of choosing 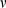 on recovered monolayer stresses, we recomputed the stress field using 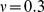 and then compared the two stress fields.

To assess the effect of heterogeneity of 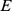, we first considered the monolayer with homogeneous elastic properties where 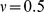 and computed the local average normal stresses. Recognizing that the cell stiffness is closely related to the cytoskeletal stress [31]–[33], we amplified this effect by taking the map of average normal stress to represent the non-uniform distribution of 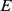, and then recalculated the stress field. We then compared the stress field recovered using homogeneous 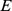 with the stress field recovered using heterogeneous 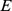.

#### Effects of boundary conditions

To quantify boundary artifacts, as in the approach used by Tambe et al. [22], we computed stresses using two different methods. In the first method, stresses were computed by solving the equations of equilibrium over the entire island; we call this case 1 (the gold standard). In the second method, stresses were computed by following the approach used by Tambe et al. [22], i.e., solving the equations of equilibrium only in the region of interest that is bounded by optical edges on three sides; we call this case 2 (Fig. 1c). The effect of the assumed boundary conditions was quantified by comparing the recovered stresses recovered from case 2 with those from the artifact free stresses computed from case 1.

### Numerical assessments

#### Propagation of the boundary artifacts

To analyze further how boundary perturbations and associated artifacts propagate into the region of interest, we imposed sinusoidal perturbations at optical edges. For example, along the right optical edge in Fig. 1c, we replaced the condition of zero normal displacements, 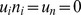, with the condition of sinusoidal fluctuations, 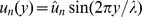, where 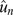 is the amplitude, 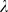 is the wavelength of the perturbation, and 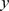 is the distance along the optical edge, with all other boundaries unchanged. We did so for wave lengths 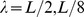. A similar set of calculations were performed with shear stress perturbations at the optical edge, 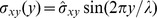, where 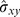 is the amplitude of the perturbation. Each of the perturbations, induced stresses in the monolayer. Spatial variation of these induced stresses is then plotted as a function of distance from the optical edge (Supporting Information S6 in File S1).

Two other monolayer geometries are of interest. The first is the monolayer bounded by two optical edges separated by two free edges (Fig. S2a in File S1). The second is the monolayer bounded by optical edges on all four sides (Fig. S2b in File S1). These cases are treated in Supporting Information S4 in File S1.

## Results

### Analytic Results

#### Scaling analysis of out-of-plane tractions and their effects

There are at least three important length scales in the problem. (1) The lateral extent of in-plane traction fluctuations 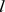, quantified by the inverse of a typical wavenumber 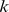 in Fourier space or an autocorrelation length in real space; (2) substrate thickness 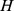; and (3) monolayer height 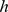. The importance of the relationship of substrate thickness to lateral extent of traction fluctuations (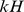 or 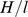) has been dealt with before [34], [35].

The remaining dimensionless number is 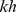, or, equivalently 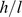. It is clear that if the monolayer height is large compared to length scale for traction fluctuations, then nothing in the 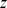 direction can be neglected. This might be the case for an isolated cell that is cuboidal or rounded up with stress fibers draped over a big nucleus. In this regime, errors associated with measuring in-plane displacements alone cannot be neglected, and are not treated here.

In the case of extended monolayers, by contrast, geometry alone gives bounds on the errors. Suppose there are true traction vectors of unit magnitude acting at two remote points separated by 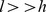; their angle 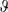 with respect to the 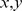 plane is at most 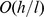. Since the recovered 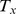 and 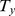 carry a factor of 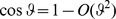, it follows that they differ from their true values only at second order in the ratio 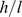. 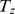 carries a 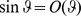 dependence, implying an error that is first order in 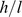.

If, for example, a typical lateral scale is 100 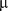
m and the monolayer cell height is 10 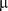
m, then the geometric arguments above imply errors on the order of 1% for in-plane tractions and 10% for out-of-plane tractions. The ratio of out-of-plane to in-plane traction would then be of order 0.1, consonant with the findings of Hur et al. [28], who reported 0.37.

#### Exact analysis of out-of-plane displacements and their effects

If 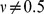, then neglecting the non-zero effects of the 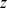 component of displacement, 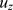, introduces errors in 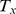 and 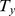. To quantify these errors we have extended the restricted two dimensional work of Butler et al. [36] to three dimensional space. A traction point source of unit force at the origin induces displacements at a point 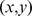 on the 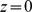 surface of a semi-infinite medium, given by the classical Boussinesq solution 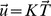, where
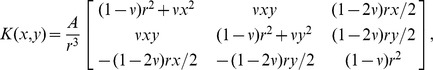


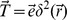
 is the point source of traction, 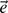 is a (3 dimensional) unit vector, and 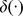 is the Dirac delta function. The prefactor is given by 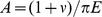, where 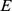 and 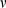 are Young's modulus and Poisson's ratio of the substrate. It is important to note that the classical Boussinesq solution is valid only when gradients of the displacements (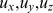) are small.

In Supporting Information S2 (in File S1), we show that in two-dimensional Fourier space, this becomes
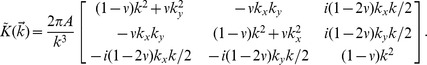
We use this formulation to quantify the errors in the recovered tractions attributable to neglecting out-of-plane displacements. As there is no intrinsic or independent length scale or preferred direction, it suffices to examine in detail the behavior for, say, 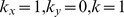, in which case we have
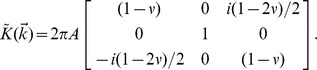
The recovery of tractions is then given by 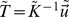, where
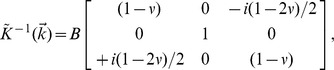
and where 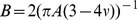.

**Figure 2:**
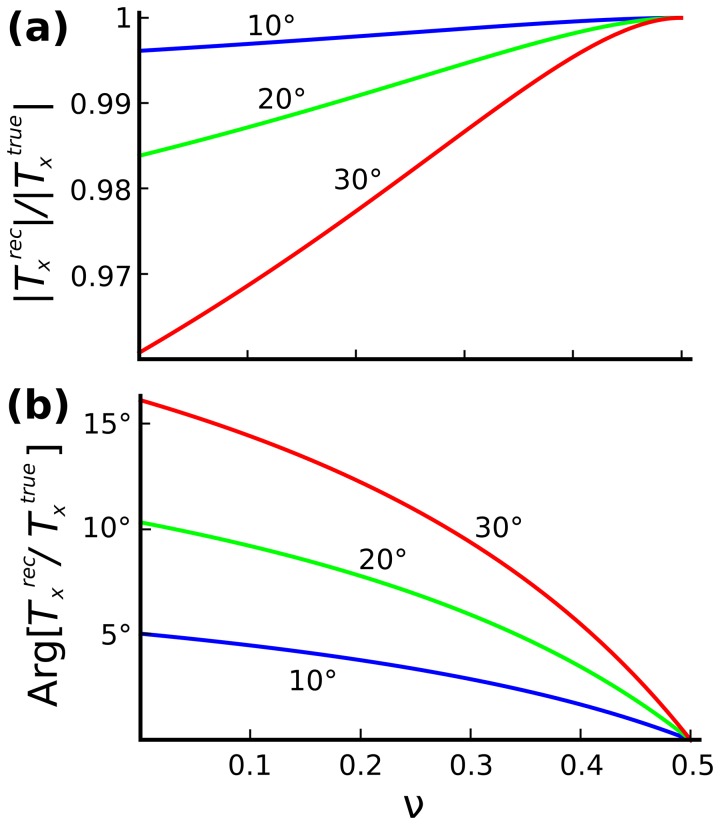
Accuracy of in-plane tractions as a function of Poisson's ratio when out-of-plane components of displacements are neglected. 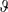
 denotes the angle of the displacement vector relative to the 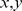 plane. (**a**) Ratio of recovered in-plane traction to true in-plane traction. (**b**) Error in the phase of the recovered in-plane traction (degrees).

We now consider a unit displacement that has three-dimensional components 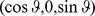, the first two being in-plane and last out-of-plane. The tractions are given by,
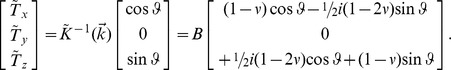
The true in-plane traction is 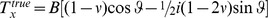. The recovered in-plane traction (neglecting the out-of-plane displacement) is 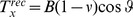. The departures from unity of the ratio 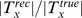 quantifies the error in magnitude, while 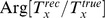 quantifies the error in phase. These quantities are plotted in Fig. 2.

When 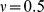, these errors vanish as expected. However, even when 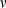 departs from 0.5 substantially, the error in recovered magnitude remains remarkably small (second order in 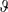) and the error in phase also remains small (first order in 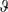). This behavior parallels the geometric argument above.

For example, taking an extreme value for polyacrylamide gels [37], 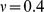, and taking 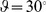, the error in magnitude would be less than 1% and the error in phase would be approximately 5°.

Finally, we remark that for substrates of finite thickness, when the tractions are smoothly distributed over distances larger than gel thickness, the substrate deformation approximates pure uniform shear, and in this case Poisson's ratio in the substrate is irrelevant.

### Experimental Results

#### Case 1: The isolated cell island

**Figure 3:**
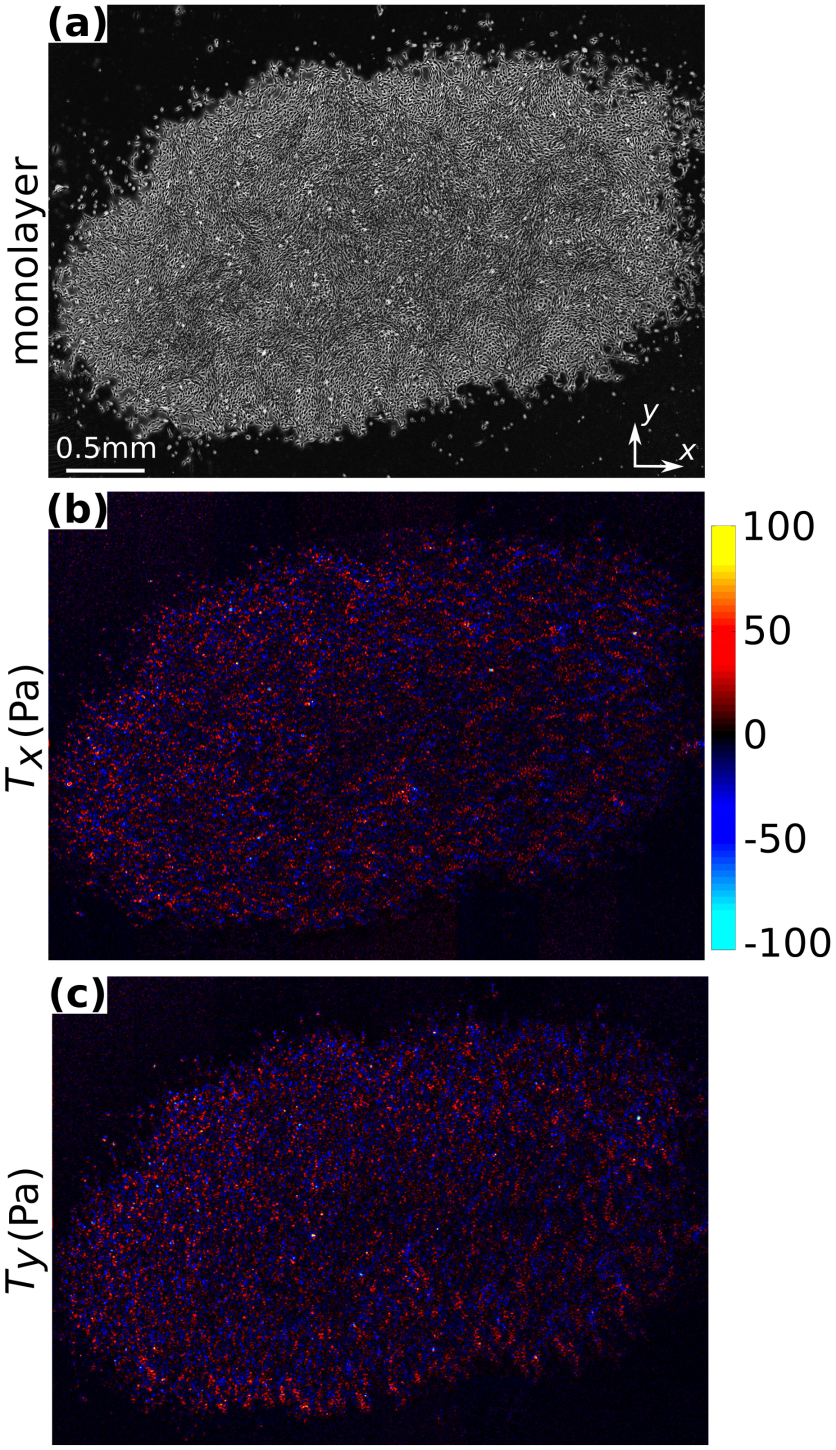
Substrate tractions for RPME cell island. (a) Island of RPME cells. (**b**,**c**) Two components of tractions, 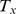 and 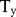 respectively, applied by these cells on the substrate. Size of the cell island: 4.2 mm×2.6 mm.

**Figure 4:**
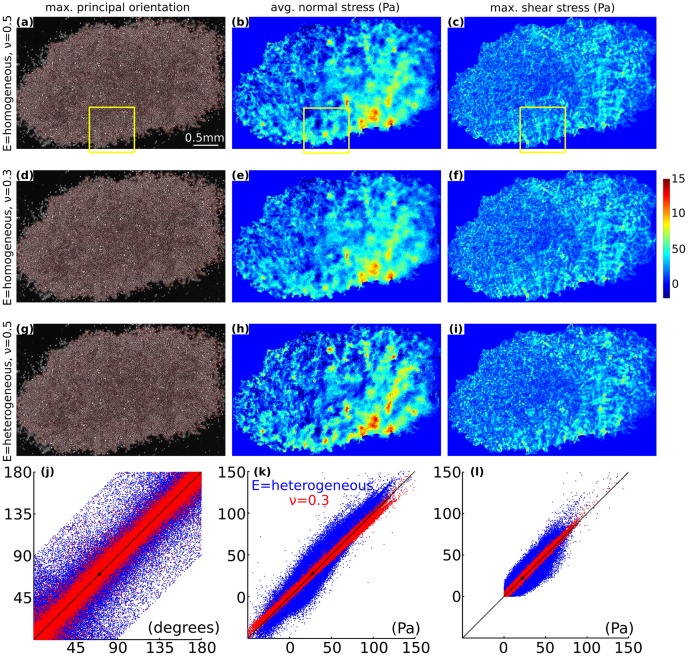
Sensitivity of the recovered monolayer stresses to change in the assumed elastic properties of the monolayer. (**a**) Map of maximum principal orientation (for enlarged version of this image, see Supporting Information S8; Figs. S11–13; in File S1), (b) map of average normal stress, and (c) map of maximum shear stress obtained by assuming monolayer elastic properties to be homogeneous with 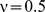. (d–f) The stress maps with 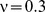 instead. (g–i) The stress maps when 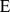 is heterogeneous with 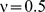, here 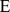 was assumed to be proportional to the map of the average normal stress (**b**). (**j**) Scatter plots for maximum principal orientation where, red points quantify effect of 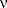 on the maps (a) and (d), and blue points quantify effect of heterogeneity of 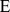 on the (a) and (g). (k) Scatter plots for average normal stress, (l) scatter plots for maximum shear stress. Regression parameters for a straight line fit, 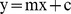 in (j–l): blue points, (j) 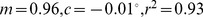, (k) 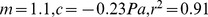, and (l) 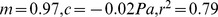; red points, (j) 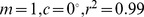, (k) 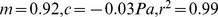, and (l) 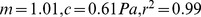.

In an island of rat pulmonary microvascular endothelial (RPME) cells, tractions demonstrated extreme spatial fluctuations (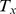, Fig. 3b; and 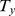, Fig. 3c). These fluctuations are comparable to those previously reported [22], [27]. Using these traction fields together with Eqs. 1 and 2, and assuming the monolayer elastic properties to be homogeneous and incompressible (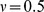), the resulting intercellular stress landscape demonstrated the same characteristic ruggedness as observed previously by Tambe et al. [22] (Fig. 4a–c).

When we reduced the assumed value of 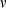 from 0.5 to 0.3, the resulting stress landscape changed relatively little (Fig. 4d–f). Similarly, when we introduced dramatic heterogeneities in 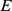 by assuming that 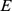 is proportional to the average normal stress (Fig. 4b), again the changes were modest (Fig. 4g–i). To quantify these differences, we created scatter plots using stresses from every grid point. On the 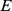-axis we plotted stresses computed for the monolayer where 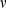 is 0.5 and 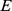 is homogeneous, and on the 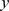-axis we plotted stresses computed for the monolayer under the other two situations. Accordingly, Fig. 4j–l represent sensitivity of stresses to the choice of 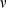 (red points) and to the heterogeneity of 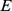 (blue points).

When 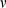 was reduced to 0.3, the three stress measures (average normal stress, the maximum principal orientation, and the maximum shear stress) each tracked closely the line of identity and showed little variation (Fig. 4k–l, red points). Unlike changes associated with the magnitude of 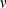, changes associated with the heterogeneity of 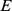 were somewhat greater (Fig. 4j–l, blue points). For average normal stress, the relationship was slightly sigmoidal (Fig. 4k, blue points); at low magnitudes the stresses were underestimated and at high magnitudes the stresses were overestimated. For maximum principal orientation, the points were symmetrically scattered around the line of identity (Fig. 4j, blue points). For maximum shear stress, the points were spread asymmetrically about the line of identity (Fig. 4l, blue points); at high magnitudes the stresses were overestimated.

Across all instances examined, the correlation coefficient 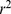 was no smaller than 0.79; the slope 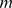 was close to unity (0.92 to 1.1); the intercept 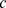 was (−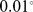 to 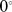) for orientation of maximum principal orientation, and (−0.23 Pa to 0.61 Pa) for average normal stress and maximum shear stress.

#### Case 2: Subsystem bounded by optical edges on three sides

**Figure 5:**
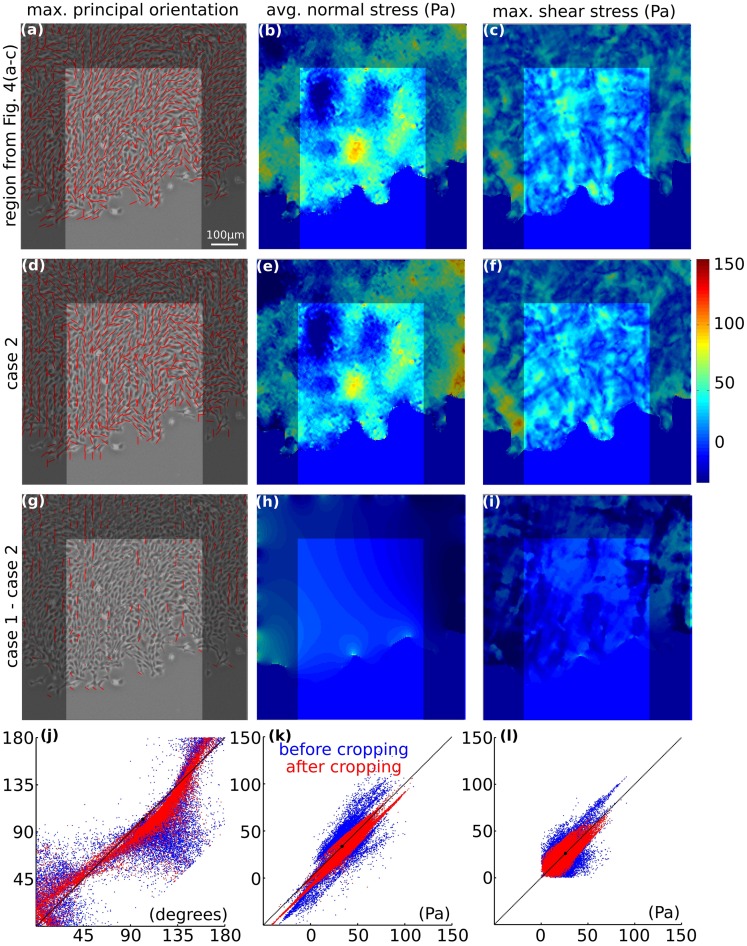
Influence of the optical edges on the monolayer stresses recovered for case 2. Maps of (**a**) maximum principal orientation, (b) average normal stress, and (c) maximum shear stress extracted from the region of interest (Fig. 4a–c, yellow rectangle). (d–f) Stress map obtained by limiting the solution of equilibrium equations to the region of interest. (g) Map of difference between (a) and (d). (h) Map of (**b**) minus (**e**). (i) Map of (**c**) minus (**f**). The grey band in (a–i) represents cropped region; width of this region was 20% of the length of the optical edge. (**j**) Scatter plots of maximum principal orientations for quantitative comparison between (a) and (d). For the blue points cropped region was included, for the red points cropped region was exclude. (k) Scatter plots for average normal stress, (l) scatter plots for maximum shear stress. Regression parameters for a straight line fit, 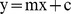 in (j–l): blue points, (j) 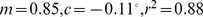, (k) 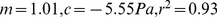, and (l) 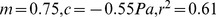; red points, (j) 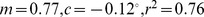, (k) 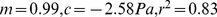, and (l) 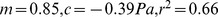. Size of the region of interest is 830 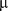
m
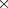
830 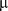
m.

Compared to the gold standard (Fig. 5a–c), the stresses computed from case 2 were systematically different (Fig. 5d–f). The stresses away from the optical edges were closely similar, whereas the stresses close to the optical edges were appreciably different (Fig. 5g–i). Moreover, as proposed by Tambe et al. [22], the differences in the stresses were largely limited to a narrow band whose width is 20% of the length of the optical edge, depicted by the grey band in Fig. 5a–f.

When the entire stress map was compared, the agreement between the gold standard and stresses from case 2 was weak, as expected (Fig. 5j–l, blue points). But when the cropped maps (which excluded the boundary region depicted by the grey band) were compared, the agreement between stresses was far better (Fig. 5j–l, red points). Nonetheless, average normal stresses were slightly underestimated (Fig. 5k, red points), the maximum principal orientations were slightly biased along a diagonal of the monolayer geometry (Fig. 5j, red points), and the maximum shear stresses were rather weakly correlated (Fig. 5l, red points).

Across all instances examined, in the region away from the optical edge, the correlation coefficient 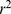 was no smaller than 0.66; the slope 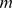 was in the range 0.77 to 0.99; the intercept 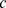 was (−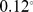 to −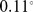) for orientation of maximum principal orientation, and (−5.5 Pa to −0.39 Pa) for average normal stress and maximum shear stress.

### Numerical Results

#### Propagation of the boundary artifacts

**Figure 6:**
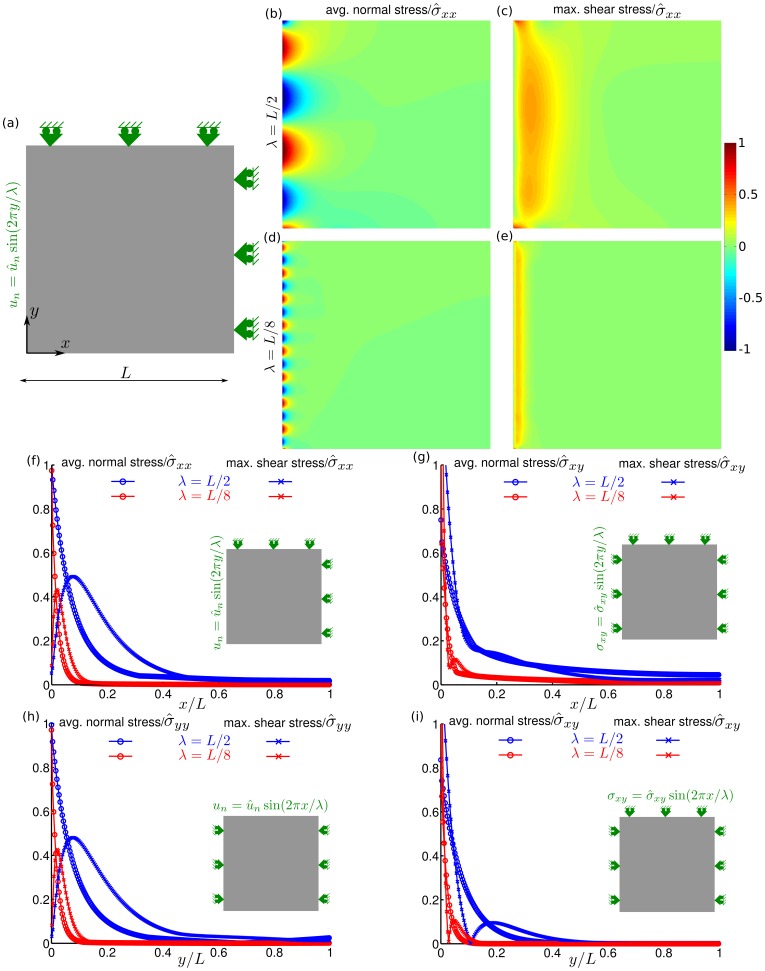
Propagation of boundary artifacts away from the optical edge. (a) A thin sheet subjected to sinusoidal perturbations in normal displacements 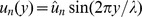 at one edge, and 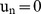 at two other edges. (b) Map of average normal stress, and (c) map of maximum shear stress when 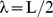. (d–e) The stress maps when 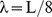. (**f**) Decay of dominant Fourier mode in the stresses induced by the boundary conditions shown in the inset. Blue curves correspond to 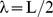, and red curves correspond to 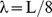. The curves marked with circle represent the induced average normal stress, and the curves marked with cross represent the induced maximum shear stress. (g–i) Decay curves of the stresses induced by boundary conditions shown in the inset. At all the boundaries along appropriate axis the natural boundary conditions, i.e. boundary stress = 0 are not mentioned but they are implied. The stresses in (b–f) are normalized with the amplitude of induced normal stress 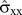 at the perturbed edge, the stresses in (g,i) are normalized with amplitude of imposed shear stress 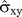, and the stresses in (h) are normalized with the amplitude of induced normal stress 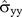 at the perturbed edge.

First, we considered an optical edge adjacent to the free edge, and imposed boundary perturbation comprising sinusoidal normal displacements 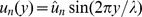, where 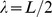 (Fig. 6a). These normal displacements induced average normal stress which decayed monotonically with distance from the boundary (Fig. 6b, and Fig. 6f, blue lines marked with circle); when the wavelength of the perturbation was smaller the decay was faster (Fig. 6d, and Fig. 6f, red lines marked with circle). Indeed, the decay length in each case was comparable to the wavelength of perturbation. While the decay of average normal stress was monotonic, the decay of maximum shear stress was not monotonic (Figs. 6c,e; and Fig. 6f lines marked with cross). Finally, when the perturbed edge was the optical edge away from the free edge, the stresses were similar (Fig. 6h).

Next, we considered the boundary perturbations comprising sinusoidal shear stress 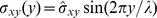. Unlike the normal displacements perturbations, the shear stress perturbations induce stresses whose rate of decay depends on choice of the optical edge. When the perturbed edge was the optical edge away from the free edge, the induced stresses decayed faster (Fig. 6i, Supporting Information S5; Fig. S8; in File S1).

Analysis of case 3 and 4 produced results that were qualitatively similar to those of the analysis of case 2 (Supporting Information S4; Figs. S3–S7 and Fig. S14; in File S1).

## Discussion

Our principal findings are these. For in-plane traction recovery and for in-plane monolayer stress recovery, the contributions of out-of-plane components of tractions and displacements are negligible. Second, optical edges cause artifacts that are mostly confined to regions near the optical edge but are otherwise homogeneous and small. Third, assumptions of homogeneity and incompressibility of the monolayer material cause artifacts that are also small. Finally, characteristic features of the recovered stress maps that underlie plithotaxis are insensitive to these artifacts, thus demonstrating that the observation of plithotaxis is robust. Below we discuss in greater detail monolayer stress recovery, associated findings, and their implications.

### Measurement of out-of-plane tractions and displacements is not necessary

Recent reports have asserted that three dimensional methods are necessary because the out-of-plane tractions or displacements can be sufficiently large to invalidate tractions and monolayer stresses recovered from two-dimensional methods [28], [29]. To assess the validity of this assertion we address three key questions.

First, to what degree do out-of-plane displacements, which are unmeasured in MSM, impact recovery of in-plane tractions? When the substrate is incompressible (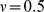) and thick (Supporting Information S7; Fig. S10; in File S1), out-of-plane displacements and in-plane tractions become decoupled [35], [38]. In that case errors attributable to ignoring out-of-plane events are precisely zero. Accordingly, in that case both two-dimensional and three-dimensional traction algorithms will recover in-plane tractions that are identical. Remarkably, even when Poisson's ratio departs substantially from 0.5 and the out-of-plane displacements are extreme, analysis shows that the errors in both magnitude and phase of the recovered in-plane tractions remain negligible (Fig. 2).

Second, are the recovered in-plane tractions insensitive to the difference between cells adhering tightly versus loosely to one another, as asserted by [29] Notbohm et al. [29]? This assertion stands in contrast with previously reported experimental data obtained using the very same cell type (MCF10A) and demonstrating the sensitivity of MSM to distinguish between control and preparations where cell-cell junctions were disrupted with cadherin antibodies ([22] [Fig. 4]). We reason that the contrary conclusion of Notbohm et al. [29] follows from a very specific feature of their model. In particular, Notbohm et al. [29] modeled the interaction between cells and substrate by elastic springs normal to the cell-substrate plane. In such a model, to first order there is no shear stress (i.e. in-plane substrate tractions) applied by the cells on the substrate. By construction, therefore, only in those springs that depart from first order strain will there be any transmission of in-plane tractions, and this occurs preferentially at the island boundary.

Third, to what degree do nonzero out-of-plane displacements and out-of-plane tractions affect the recovered in-plane monolayer stresses? If out-of-plane tractions 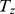, and displacements 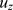 are neglected, the accuracy of the in-plane monolayer stresses would, in principle, be affected in two ways. (i) Neglecting the out-of-plane components can have an indirect effect on the monolayer stresses via the errors introduced in the in-plane tractions. However, Fig. 2 shows that the effect of neglecting 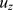 on the recovered in-plane tractions is remarkably small. Hence, the indirect effects on the monolayer stresses are also expected to be small. (ii) Neglecting the out-of-plane components can have a direct effect on the monolayer stresses. In the limit of classical thin plate theory, 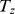 will induce bending moments in the monolayer, whose contribution to 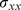, 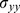, and 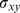 scale as 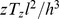, where 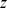 has its origin at the mid-plane of the monolayer. The thickness average of this contribution is, however, identically zero. In connection with the distribution of three-dimensional tractions, Notbohm et al. ([29]; Fig. 6a) propose a model, according to which both 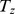 and associated bending moments are largely confined to the monolayer boundary. Near the boundary, in the limit of classical plate theory the thickness averaged monolayer stresses are negligible. Away from the boundary, the monolayer stresses have even higher accuracy. For example, beyond a distance proportional to the height of the monolayer, according to St. Venant's principle, the bending moments and, therefore, the errors in monolayer stresses will be negligible (Note that this argument is not restricted to the classical thin plate theory). Taken together, the three-dimensional traction model of Notbohm et al. [29] suggests that monolayer stresses recovered by neglecting 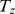 would have associated errors that are negligible.

Moreover, it has been suggested in several reports that the problem of intercellular stress recovery, given the traction field, is mathematically intractable [19], [28], [39]. It is true that there are boundary artifacts caused by optical edges which introduce slowly varying but unknown additive terms. Measurements from an island, which has no optical edge, suffer from no such artifact, however, and, as such, monolayer stresses can be determined uniquely. Further, direct comparison of stress distributions computed for an entire island compared with stress distributions computed for a sub-region of interest bounded by optical edges (assuming appropriate boundary conditions) show only modest differences after cropping (Figs. 5k, S3k, S5k). In summary, for isolated islands the problem of recovery of stress distributions is mathematically tractable; for monolayers extending outside the field-of-view, the stress distributions can be estimated accurately using MSM with suitable care.

### Boundary artifacts are confined largely to the boundary

*Ad hoc* boundary conditions at the optical edge introduced systematic artifacts in the recovered distribution of monolayer stresses, especially near optical edges. Near the optical edge, these artifacts were heterogeneous and large (Fig. 5g–i, region shaded in grey, and Fig. 5j–l, blue points). However, away from the optical edge these artifacts were largely homogeneous and quite small (Fig. 5g–i, non-shaded region, and Fig. 5j–l, red points). These findings confirm and extend the analysis of Tambe et al. [22].

Subsequent to Tambe et al. [22], Hur et al. [28] analyzed a square monolayer region bounded on all four sides by optical edges. At the optical edges, instead of assuming symmetric boundary conditions (see Introduction, Boundary Conditions and [22]), Hur et al. [28] assumed stress-free boundary conditions, which mechanically uncouples the region of interest from the rest of the monolayer. To avoid unknown influences of cells outside the optical field-of-view, Hur et al. [28] then cropped their stress maps but much less so (by a factor of six) than did Tambe et al. [22]. Monolayer stresses computed with the cropping and boundary conditions of Hur et al. [28] had a poor correlation 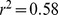 with artifact-free stresses computed from an entire island. By contrast, monolayer stresses obtained using the cropping and boundary conditions of Tambe et al. [22], were tightly correlated 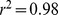 with stresses computed from the entire island. (Fig. S5k, red points, in File S1).

### Artifacts attributable to material properties are small

On the one hand, a recent study [40] makes the striking observation that across a monolayer the cell stiffness is extremely homogeneous. On the other hand, in isolated cells we have previously established a direct proportionality between cytoskeletal stress and cytoskeletal stiffness [31]–[33], which we assumed here. As regards heterogeneity of elastic cellular properties within a monolayer, therefore, these extreme cases would seem to bound the plausible possibilities.

If monolayer material properties are homogeneous, then stress recovery using MSM is unaffected. If the properties are heterogeneous (Fig. 4b), recovered stresses at high stress magnitudes were slightly underestimated, and at low stress magnitudes were slightly overestimated (Fig. 4k, blue points). Therefore, although the recovered stress landscape would be slightly flattened, the ruggedness of the stress landscape remains a robust finding. The assumption of incompressibility caused no such artifacts, however (Fig. 4j–l, red points). Moreover, the prediction of local cell orientation by local maximum principal stress orientation was robust (Supporting Information S3; Fig. S1; in File S1). As such, recovered stresses depended upon assigned material properties, as expected, but the magnitude of these effects was unexpectedly small.

Although movement of the epithelial sheet has been shown to respond to the viscosity of the substrate [41], here we take the substrate to be simply and linearly elastic, as in traditional polyacrylamide and PDMS. As for the cell monolayer itself, most mathematical models of development assume that the dominant mechanical stress in cells comprising the monolayer is viscous on the slow time scales relevant to migration [42]. Recent experimental data demonstrate, to the contrary, that the dominant mechanical stress in cells comprising the monolayer is very nearly elastic [23]. Moreover, since the monolayer stress recovery rests not on dynamic but on instantaneous balance of the substrate tractions, neglecting viscosity of the monolayer has no associated artifact.

### The observation of plithotaxis is robust

According to the principle of plithotaxis, the local orientation of cell migration and the local orientation of maximum principal stress are strongly associated [22]. Might the artifacts addressed here impact this finding? The maximum principal orientation was found to be largely insensitive to the artifacts addressed here (Figs. 5j, S3j, and S5j), thus leaving the finding of plithotaxis unaltered.

The association between the stress and the motion is related to the local stress anisotropy; the higher is the anisotropy, the tighter is the association [22]. Although the finding of plithotaxis was insensitive to the artifacts addressed here, the anisotropy of the stress (i.e. the maximum shear stress) was, however, affected by the boundary artifacts (Fig. 5l, after data cropping, 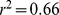). Nonetheless, in the regions where anisotropy was highest and lowest, the degree of anisotropy was largely preserved (data not shown). Hence, the observation that higher stress anisotropy lead to tighter mechanical guidance is also robust.

Based upon this evidence and these arguments, we agree that out-of-plane tractions may be present and are of some interest. However, out-of-plane tractions are ordinarily uncoupled from, and therefore have no appreciable effects upon, recovered in-plane stresses.

## Conclusions

For the cellular monolayer *in vitro*, MSM maps thickness-averaged in-plane components of the complete two-dimensional stress tensor [22]. Although MSM ignores out-of-plane stress components, it recovers in-plane stress components with negligible error and without the requirements for confocal microscopy and computational resources required for three-dimensional reconstructions [28], [29]. MSM is not subject to the additional errors that might be attributable to experimental imprecision in resolving 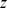-displacements, moreover, or to computational error in three-dimensional finite element analysis. Stress recovery using MSM is simpler than three dimensional approaches and is insensitive to those artifacts that have been identified.

## Supporting Information

File S1 **Supporting Information S1–S8.** Details of experimental and mathematical approach and accuracy assessment for two other monolayer geometries of interest. The supporting information contains several topics which includes, protocol for mapping stresses (S1), Fourier representation of three dimensional Boussinesq solution (S2), data for alignment between maximum principal orientation and cell orientation (S3), accuracy assessment for two other monolayer systems of interest (S4), effect of adjacent free edges on rate of decay of boundary artifacts (S5), procedure for mapping decay of boundary artifacts (S6), effect of substrate thickness on substrate tractions (S7), and enlarged images of selected results (S8).
(PDF)
