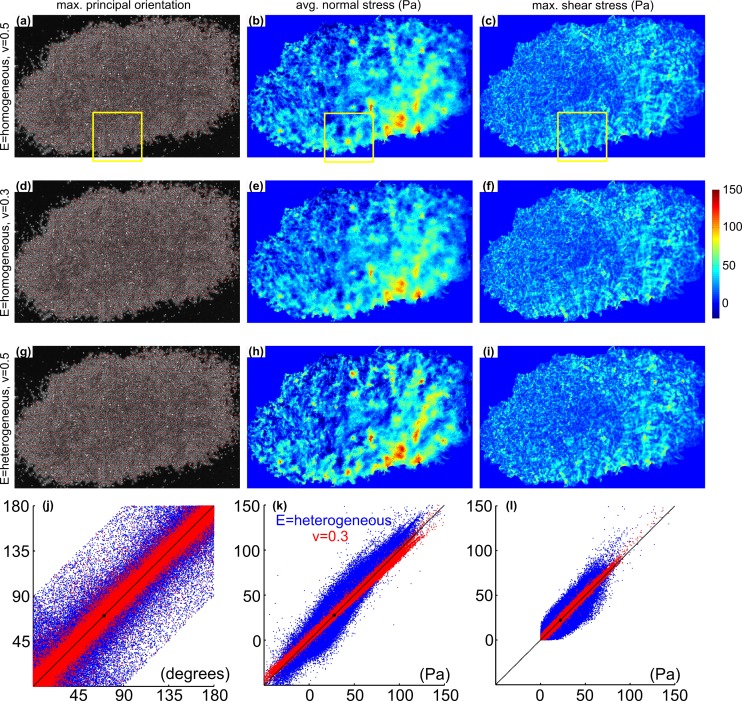# Correction: Monolayer Stress Microscopy: Limitations, Artifacts, and Accuracy of Recovered Intercellular Stresses

**DOI:** 10.1371/annotation/d29aed40-7667-4901-8008-ef473c363216

**Published:** 2014-01-17

**Authors:** Dhananjay T. Tambe, Ugo Croutelle, Xavier Trepat, Chan Young Park, Jae Hun Kim, Emil Millet, James P. Butler, Jeffrey J. Fredberg

Figure 2, "Accuracy of in-plane tractions as a function of Poisson's ratio when out-of-plane components of displacements are neglected" is incorrect. The correct file can be found here: 

**Figure:**
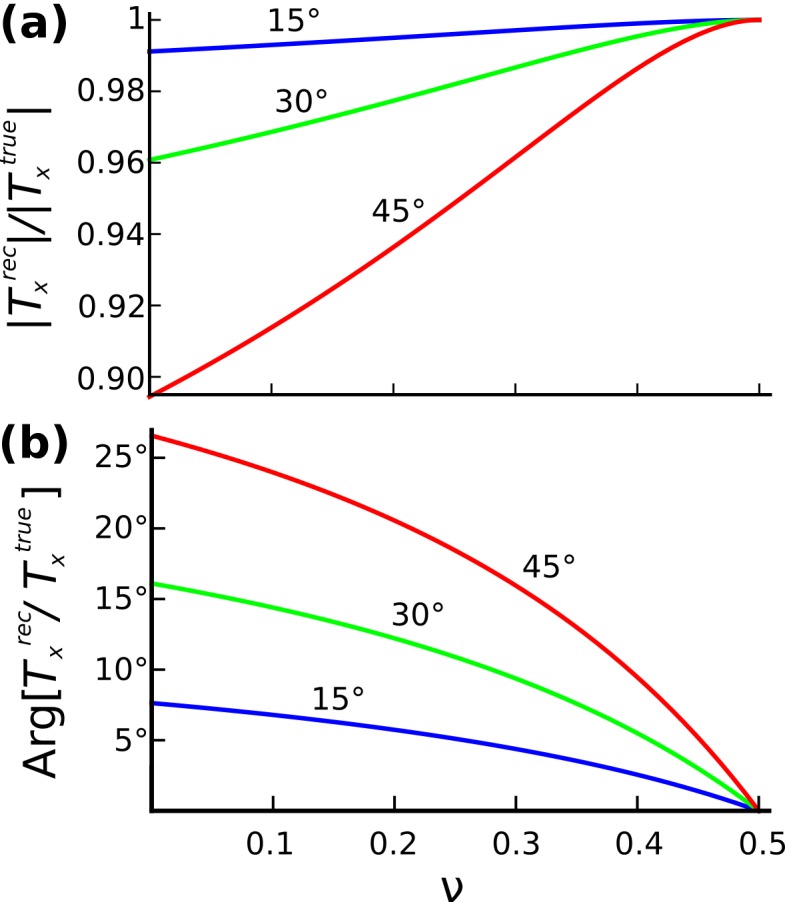


The color bar in Figure 4, "Balance of forces considered in MSM" is incorrectly displayed to range from 0 to 15Pa. Correct range is 0 to 150Pa. The correct version of Figure 4 can be found here: 

**Figure:**