# Supplementary material for: Monolayer Stress Microscopy: Limitations, Artifacts, and Accuracy of Recovered Intercellular Stresses
Source: PLoS One. 2013 Feb 28;8(2):e55172. doi: 10.1371/journal.pone.0055172 (PMC3585344; doi:10.1371/journal.pone.0055172)
Supplement: File S1 — Supporting Information S1–S8. Details of experimental and mathematical approach and accuracy assessment for two other monolayer geometries of interest. The supporting information contains several topics which includes, protocol for mapping stresses (S1), Fourier representation of three dimensional Boussinesq solution (S2), data for alignment between maximum principal orientation and cell orientation (S3), accuracy assessment for two other monolayer systems of interest (S4), effect of adjacent free edges on rate of decay of boundary artifacts (S5), procedure for mapping decay of boundary artifacts (S6), effect of substrate thickness on substrate tractions (S7), and enlarged images of selected results (S8). (PDF) [file pone.0055172.s001.pdf]

ONLINE SUPPORTING INFORMATION

Monolayer Stress Microscopy: limitations, artifacts, and accuracy of recovered intercellular stresses

Dhananjay T. Tambe, Ugo Croutelle, Xavier Trepat, Chan-Young Park, Jae Hun Kim, Emil Millet, James P. Butler, Jeffrey J. Fredberg

Supporting Information S1. Protocol for mapping stresses.
The rat pulmonary microvascular endothelial (RPME) cells were a gift from Dr. Usamah Kayyali (Tufts University) [1]. For these cells, the culture protocol was similar to that described by An et al. [1], and the seeding protocol was similar to that described by Tambe et al. [2]. Gel preparation was similar to that described by Trepat et al. [3].

Acquisition of images: mosaics and tiles.
Each specimen consisted of a cell monolayer cultured on polyacrylamide gel and mounted on an inverted microscope with a motorized stage. The monolayer area was typically four times larger than the microscopic field-of-view. Each phase contrast image of an entire monolayer was acquired as an image mosaic of smaller image tiles, where the two fields-of-view of neighboring tiles were chosen to have an approximate 20% overlap. This was followed by a similar set of images of the fluorescent beads. After removing the cells with trypsin, this acquisition procedure was repeated, both for phase contrast and for the fluorescent beads.

Corrections for mosaic construction and overall stage drift.
To correct the original mosaic comprising the individual tiles for errors in motorized stage displacements, we chose small regions (approximately 96 × 96 pixels) in the middle of the overlap of pair-wise image tiles, taken sequentially from top to bottom and left to right in the original mosaic. For each tile in this sequence, we shifted the succeeding tile in both x and y, by integer pixels, such that the cross correlation of that tile with the previous set was maximized. This procedure was done separately for the phase contrast and fluorescent bead mosaics.

To correct for stage drift between two fluorescent bead mosaics, before and after trypsinization, a similar procedure was used, following [2]. Within an unstrained area (a region of the gel at least 200 μm away from the monolayer), we again chose a square region approximately 96 $\times$ 96 pixels. We then shifted the after-trypsin mosaic relative to the before-trypsin mosaic by a displacement that maximized the cross correlation between them.

Calculation algorithms.
To calculate the gel deformation from the fluorescent bead mosaics, we used the particle image velocimetry (PIV) procedure of Trepat et al. [3]. To calculate tractions, we used the Fourier-transform traction algorithm described by Butler et al. [4] and Trepat et al. [3]. To calculate monolayer stresses, we used the finite element procedure described by Tambe et al. [2].

Boundary conditions for the monolayer.
For the monolayer bounded by free edges on all sides (Fig. 3a), the entire boundary was subjected to the stress-free condition $\sigma_{ij} n_j = 0$ where n_j is the unit vector normal to the monolayer boundary. To make the boundary value problem statically determinate, we constrain rigid displacements and

rotations as follows. It suffices to pick two boundary points with the same y-coordinate, and at one to impose $u_x = u_y = 0$ and at the other to impose $\sigma_{xj} n_j = u_y = 0$. For the monolayer region bounded by optical edges on three sides (Fig. 1c), we used the boundary conditions described by Tambe et al. [2].

Supporting Information S2. Fourier representation of the three dimensional Boussinesq solution.

Butler et al. [4] solved the two dimensional inverse problem recovering tractions from surface displacements restricted to in-plane tractions and displacements. Here we extend that analysis to the three dimensional relationship between u_i, T_i and ν in Fourier space. We adopt the notation of Butler et al. [4]; let the in-plane coordinate system be given by $\vec{r} = (x, y)^T$. The response function (or Green's function) of the 3 dimensional surface displacements $\vec{u}$, given a point traction source of unit force at the origin on the $z = 0$ plane is given by the Boussinesq solution, $\vec{u} = K\vec{T}$, where $\vec{T} = \vec{e}\delta^2(\vec{r})$ is the point source of traction, $\vec{e}$ is a (3 dimensional) unit vector, and $\delta(\cdot)$ is the Dirac delta function. The matrix K is given by,

$$K(x,y) = \frac{A}{r^3}\begin{bmatrix} (1-\nu)r^2 + \nu x^2 & \nu xy & (1-2\nu)rx/2 \\ \nu xy & (1-\nu)r^2 + \nu y^2 & (1-2\nu)ry/2 \\ -(1-2\nu)rx/2 & -(1-2\nu)ry/2 & (1-\nu)r^2 \end{bmatrix}$$

where $A = (1+\nu)/\pi E$.

We now seek the 2 dimensional Fourier transform of this kernel, with the transform defined by $FT_2(f) = \int d^2\vec{r}\exp(i\vec{k}\cdot\vec{r})f(\vec{r})$. The upper 2×2 block is the two dimensional relationship of in-plane and out-of-plane tractions and displacements; its transform is given in Butler et al. [4] (typographical sign errors in the off diagonals are here corrected). The transform of K_{zz} requires $FT_2(1/r) = 2\pi/k$ (also given in [4]). It remains to compute $FT_2(x/r^2)$ and $FT_2(y/r^2)$, which unfortunately cannot be done by the methods used before. The computation does yield, however, to the following approach. Note that these two terms are the components of $FT_2(\vec{r}/r^2)$, which, being a vector, must be proportional to the only other vector in the problem, namely $\vec{k}$. So, we write $FT_2(\vec{r}/r^2) = c\vec{k}$, and consider $\vec{k}\cdot FT_2(\vec{r}/r^2) = ck^2$. In this form, we can evaluate the constant by direct calculation,

$$\begin{aligned}\vec{k}\cdot FT_2(\vec{r}/r^2) &= \vec{k}\cdot\int d^2\vec{r}\exp(i\vec{k}\cdot\vec{r})\vec{r}/r^2 = k\int_0^\infty dr\int_0^{2\pi} d\vartheta\exp(ikr\cos\vartheta)\cos\vartheta \\ &= k\int_0^\infty dr 2\pi i J_1(kr) = 2\pi i\end{aligned}$$

where we have chosen the polar origin along $\vec{k}$, used an integral representation for the Bessel function of the first kind, and its integral property of normalization. It follows that the constant $c = 2\pi i/k^2$, and so we find $FT_2(\vec{r}/r^2) = 2\pi i\vec{k}/k^2$. From this the separate x, y components are given by the k_x, k_y components of the transform.

The transform of the full three dimensional relationship between tractions and displacements is therefore given by,

$$\tilde{K}(\vec{k}) = \frac{2\pi A}{k^3}\begin{bmatrix} (1-\nu)k^2 + \nu k_y^2 & -\nu k_x k_y & i(1-2\nu)k_x k/2 \\ -\nu k_x k_y & (1-\nu)k^2 + \nu k_x^2 & i(1-2\nu)k_y k/2 \\ -i(1-2\nu)k_x k/2 & -i(1-2\nu)k_y k/2 & (1-\nu)k^2 \end{bmatrix}.$$

Supporting Information S3. Alignment between maximum principal orientation and cell orientation.
For a monolayer of elongated cells (e.g. RPME cells), Tambe et al. [2] found that monolayer stresses align with local cell orientation. Below we examine whether this alignment might be affected by the artifacts attributable to cell material properties.

In order to quantify the alignment, we define an angle β between the local maximum principal stress orientation and the local cell orientation (as these are non-directed orientations, we take $0 \le \beta \le 90°$). When the elastic properties were homogeneous and $\nu = 0.5$ (Fig. 4a), β was distributed narrowly about zero (Fig. S1a). Even when $\nu = 0.3$ (Fig. 4d) or the elastic properties were heterogeneous (Fig. 4g), β was still distributed narrowly about zero (Fig. S1b and S1c). Taken together, these distributions demonstrate that ability of the monolayer stresses to predict local cell orientation is insensitive to the artifacts attributable to the material properties.

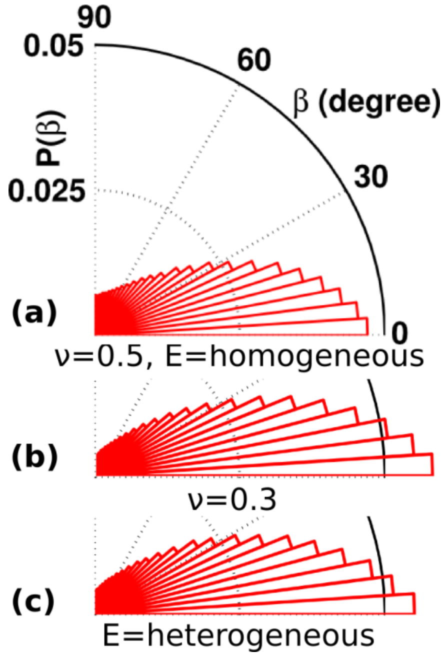

Figure S1. Cell orientation aligned with maximum principal orientation independently of cell material properties. (**a**) For the cell island (Fig. 4a), the angle β between local maximum principal orientation and local cell orientation was distributed narrowly about zero when material properties were considered to be homogeneous with $\nu = 0.5$. However, even when $\nu = 0.3$ or E=heterogeneous, the distribution of β was largely unaffected (**b**, **c** respectively).

Supporting Information S4. Two other monolayer subsystems of interest.
Case 3: Subsystem bounded by two optical edges separated by two free edges.

Recent studies of collective cell migration used a monolayer subsystem where the region-of-interest was bounded by two optical edges separated by two free edges (Fig. S2a) [5,6]. For this subsystem, stresses can be computed by solving the equations of equilibrium within the region of interest alone; we call this approach as case 3. For this case, boundary conditions were similar to those in case 2 (i.e. $u_i n_j = 0$ and $\sigma_{ij} n_j t_i = 0$ at the optical edge; and $\sigma_{ij} n_j = 0$ at the free edge). To make the boundary value problem statically determinate, we chose a point on the top left corner of the monolayer and constrained both of its displacements (i.e. $u_i = 0$).

Case 4: Subsystem bounded by optical edges on all four sides.
Most studies of endothelium use a monolayer subsystem where the region-of-interest is bounded by optical edges on all four sides (Fig. S2b) [7]. For this subsystem, stresses can be computed by solving the equations of equilibrium within the region of interest alone; we call this approach as case 4. For this case, all boundaries were subjected to $u_i n_i = \sigma_{ij} n_j t_i = 0$.

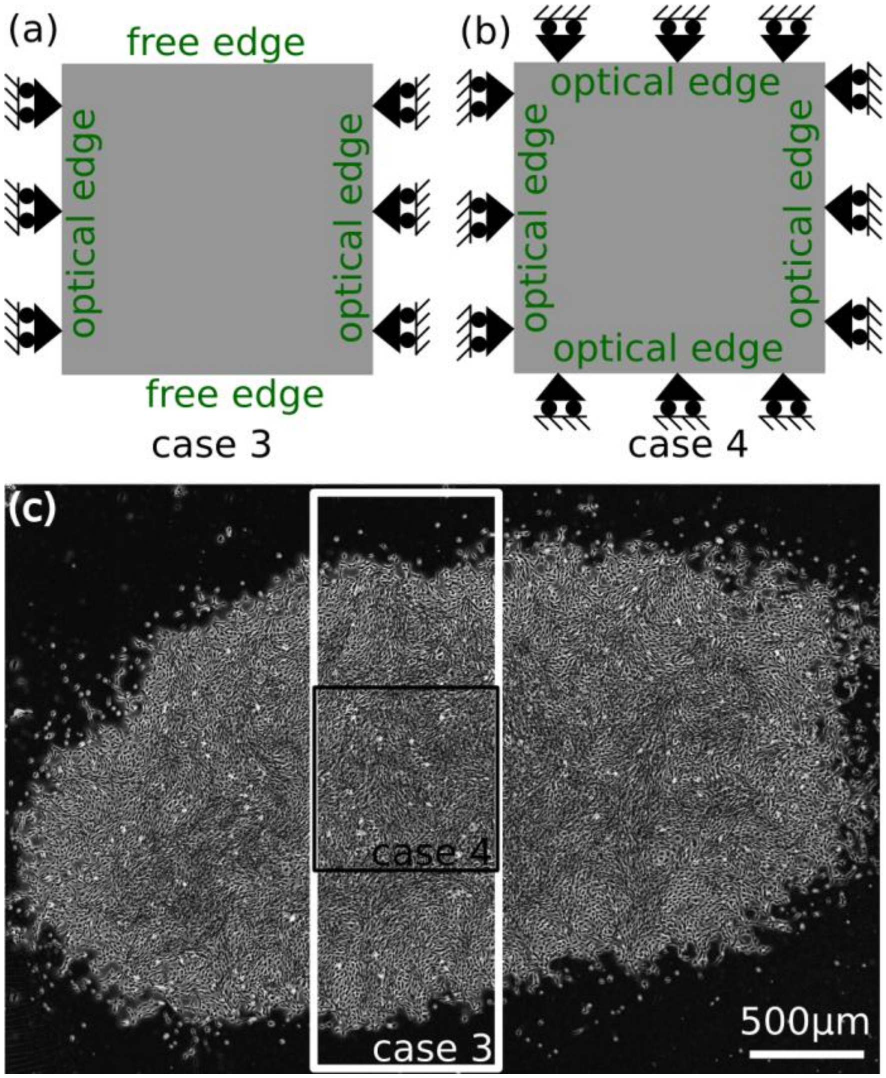

Figure S2. Two monolayer subsystems of interest. (**a**) Case 3: monolayer bounded by two free edges separated by two optical edges. (**b**) Case 4: monolayer bounded by optical edges on all four sides. (**c**) Both of these subsystems were generated from a bigger island.

Experimental results for case 3:

Artifacts attributable to the boundary conditions:

The results were qualitatively similar to those obtained for case 2 (Fig. 5).

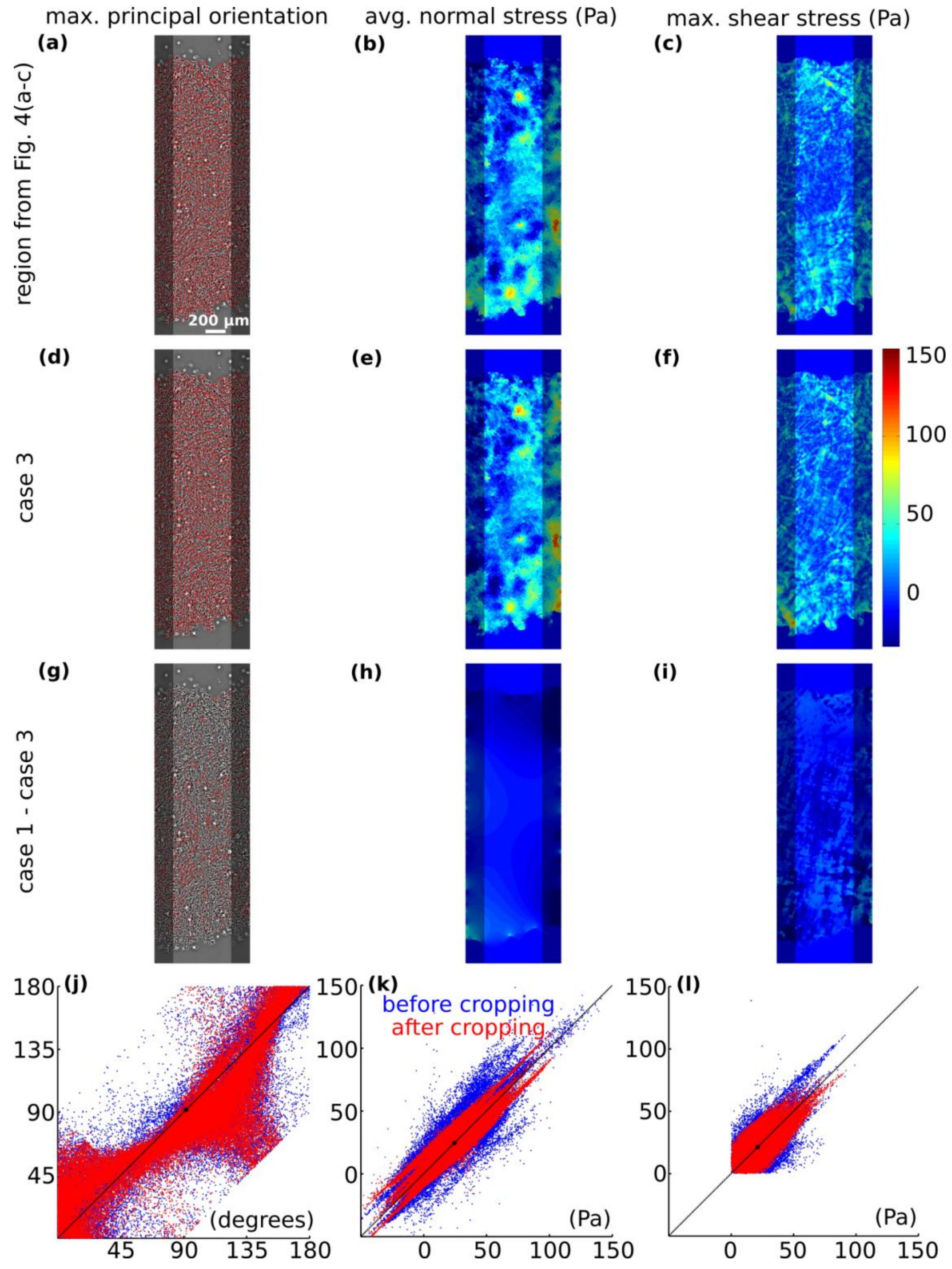

Figure S3. Influence of the optical edges on the monolayer stresses recovered for case 3. (**a**) Map of maximum principal orientation (for enlarged version of this image, see Supporting Information S8; Fig. S14), (**b**) map of average normal stress, and (**c**) map of maximum shear stress obtained over the the region of interest (Fig. S2c, white rectangle). These maps are extracted from Fig. 4a-c. (**d-f**) Stress map obtained by limiting the solution of equilibrium equations to the region of interest. (**g**) Map of difference in stress (**a**) and (**d**). (**h**) Map of (**b**) minus (**e**). (**i**) Map of (**c**) minus (**f**). The grey band in (**a-i**) represents cropped region; width of this region is same as that used in Fig. 5. (**j**) Scatter plots for maximum principal orientation that quantifies difference between (**a**) and (**d**). In blue points cropped region is included, in red points cropped region is exclude. (**k**) Scatter plots for average normal stress, (**l**) scatter plots for maximum shear

stress. Regression parameters for a straight line fit, $\delta y_i = m\delta x_i + c$ in (**j-l**): blue points, (**k**) $m = 0.78, c = -0.12°, r^2 = 0.78$, (**j**) $m = 0.91, c = 0.09Pa, r^2 = 0.87$, and (**l**) $m = 0.68, c = -0.68Pa, r^2 = 0.52$; red points, (**k**) $m = 0.78, c = -0.1°, r^2 = 0.75$, (**j**) $m = 0.95, c = 0.37Pa, r^2 = 0.82$, and (**l**) $m = 0.79, c = -0.38Pa, r^2 = 0.62$. Size of the region of interest is 830 μm × 2600 μm.

Numerical results for case 3:

Artifacts attributable to the boundary conditions:

In case 2, the relative location of optical edges and free edges is asymmetric, and as such, it has two optical edges with slightly different forms of boundary artifacts (inset in Figs. 6f and 6h). By contrast, in case 3, the relative location of optical edges and free edges is symmetric, and as such, each optical edge has a similar form of boundary artifact (inset in Fig. S4a). The decay of stresses was similar to that in case 2 where the perturbed edge was located between an optical edge on one side and free edge on other (Figs. 6f,g).

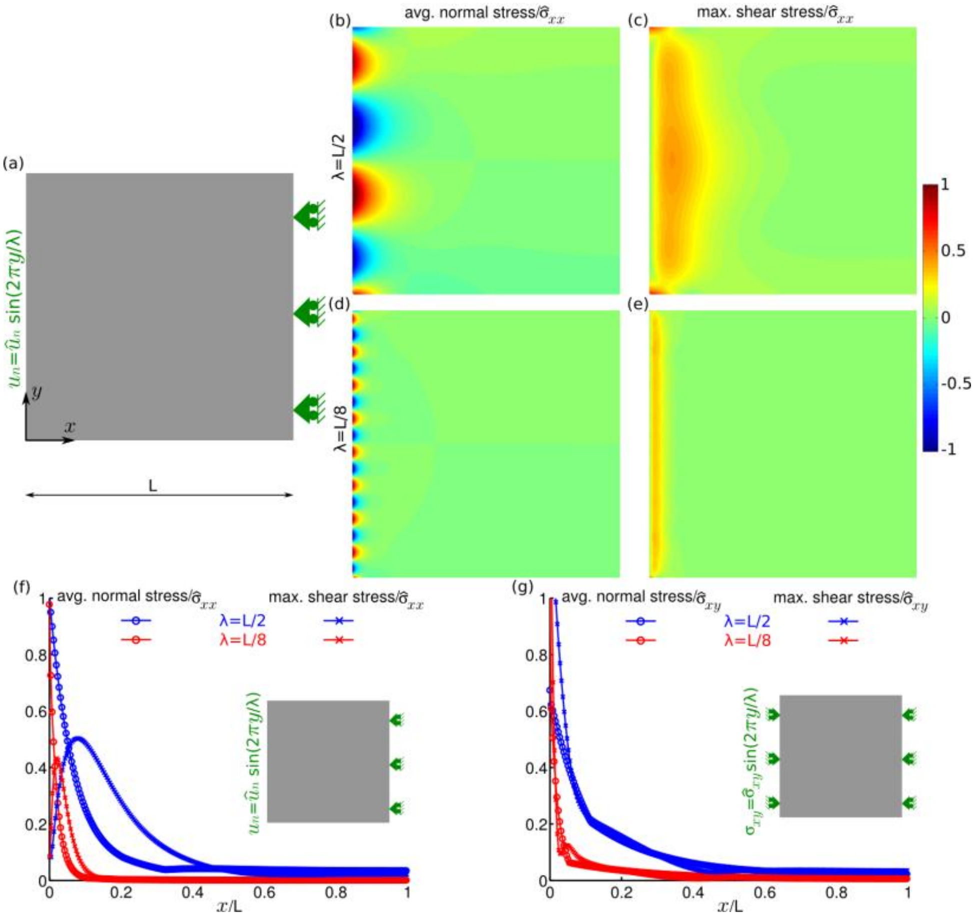

Figure S4. Propagation of boundary artifacts away from the optical edge for case 3. (a) A thin sheet subjected to sinusoidal perturbations in normal displacements $u_n = \hat{u}_n \sin(2\pi y/\lambda)$ at one edge, and $u_n = 0$ at two other edges. (b) Map of average normal stress, and (c) map of maximum shear stress when $\lambda = L/2$. (d-e) The stress maps when $\lambda = L/8$. (**f**) Decay of dominant Fourier mode in the stresses induced by the boundary conditions shown in the inset. Blue curves correspond to $\lambda = L/2$, and red curves correspond to $\lambda = L/8$. The curves marked with circle represent the induced average normal stress, and the curves marked with cross represent the induced maximum shear stress. (g) Decay curves of the stresses induced by boundary conditions shown in the inset. At all the boundaries along appropriate axis the natural boundary conditions, i.e. boundary stress=0 are not mentioned but they are implied. The stresses in (b-f)

are normalized with the amplitude of induced normal stress $\hat{\sigma}_{xx}$ at the perturbed edge, the stresses in (g) are normalized with amplitude of imposed shear stress $\hat{\sigma}_{xy}$.

Experimental results for case 4:

Artifacts attributable to the boundary conditions:

Compared with cases 2 and 3, stresses obtained from case 4 were more closely correlated with stresses obtained from the gold standard; after data cropping, $r^2 > 0.66$ for case 2, $r^2 > 0.62$ for case 3, and $r^2 > 0.84$ for case 4. By contrast, the average normal stresses obtained from case 4 had a bigger offset with the gold standard than the offsets in cases 2 and 3; after data cropping, $c = -2.58$Pa for case 2, $c = 0.37$Pa for case 3, and $c = -26.7$Pa for case 4.

In Supporting Information S5, we propose the source of better correlations in case 4.

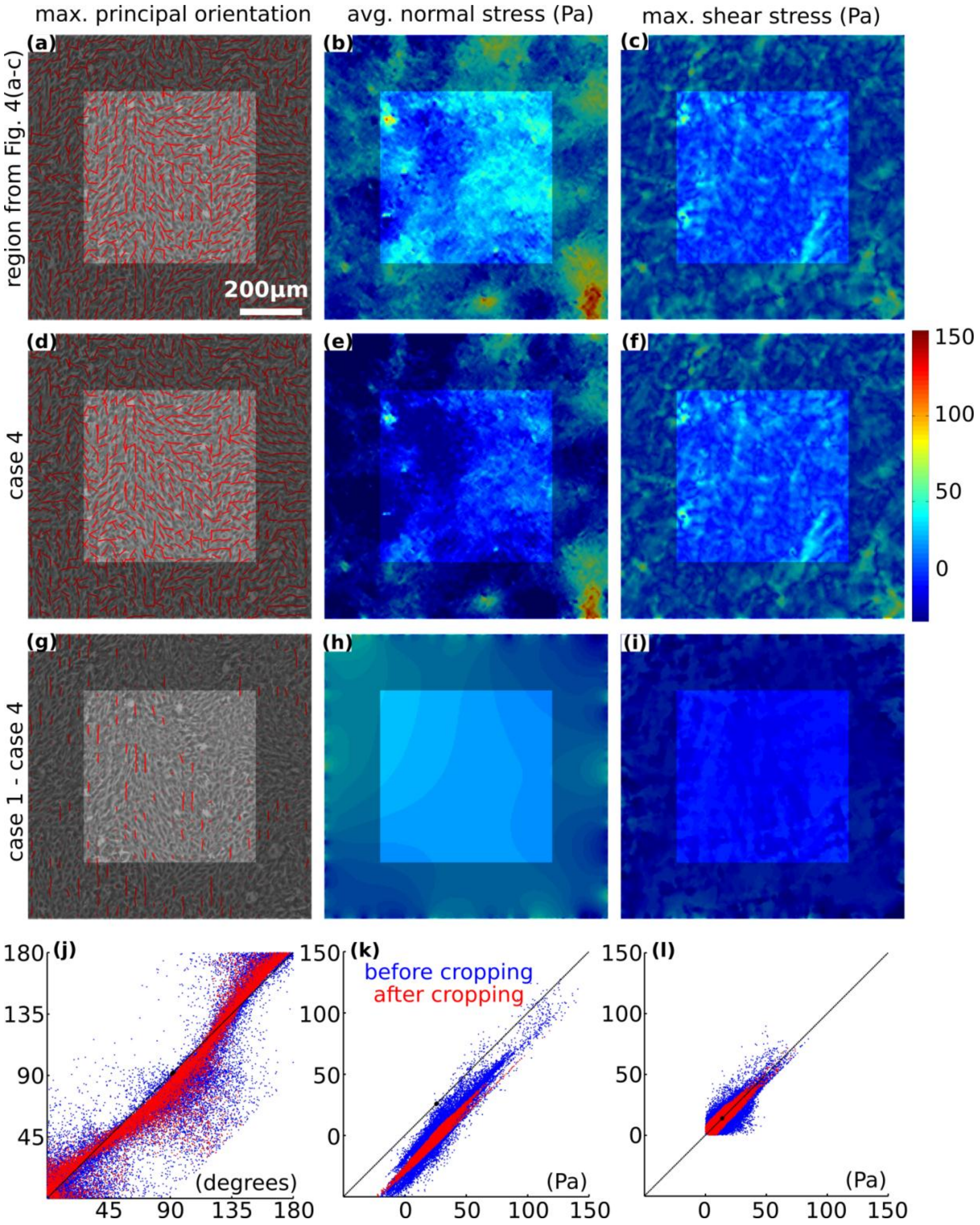

Figure S5. Influence of the optical edges on the monolayer stresses recovered for case 4. (**a**) Map of maximum principal orientation, (b) map of average normal stress, and (c) map of maximum shear stress obtained over the the region of interest (Fig. S2c, black rectangle). These maps are extracted from Fig. 4a-c. (d-f) Stress map obtained by limiting the solution of equilibrium equations to the region of interest. (g) Map of difference in stress (a) and (d). (h) Map of (**b**) minus (**e**). (i) Map of (**c**) minus (**f**). The grey band in (a-i) represents cropped region; width of this region is same as that used in Fig. 5. (**j**) Scatter plots for maximum principal orientation that quantifies difference between (a) and (d). In blue points cropped region is included, in red points cropped region is exclude. (k) Scatter plots for average normal stress, (l) scatter plots for maximum shear stress. Regression parameters for a straight line fit, $\delta y_i = m\ \delta x_i + c$ in (j-l): blue points, (j) $m = 1, c = -0.09\,°, r^2 = 0.89$, (k) $m = 1.12, c = -26.1$Pa$, r^2 = 0.92$, and (l) $m = 0.82, c = -1.04$Pa$, r^2 = 0.7$; red points, (j) $m = 1.02, c = -0.06\,°, r^2 = 0.93$, (k) $m = 1.07, c = -26.7$Pa$, r^2 = 0.98$, and (l) $m = 0.89, c = -0.25$Pa$, r^2 = 0.84$. Size of the region of interest is 830μm × 830μm.

Numerical results for case 4:

Artifacts attributable to the boundary conditions:

Case 4 has optical edges on all sides, and hence every optical edge has similar form of boundary artifact (inset in Fig. S6a). The decay of stresses was similar to that in case 2 where the perturbed edge was located between two optical edges (Figs. 6h,i).

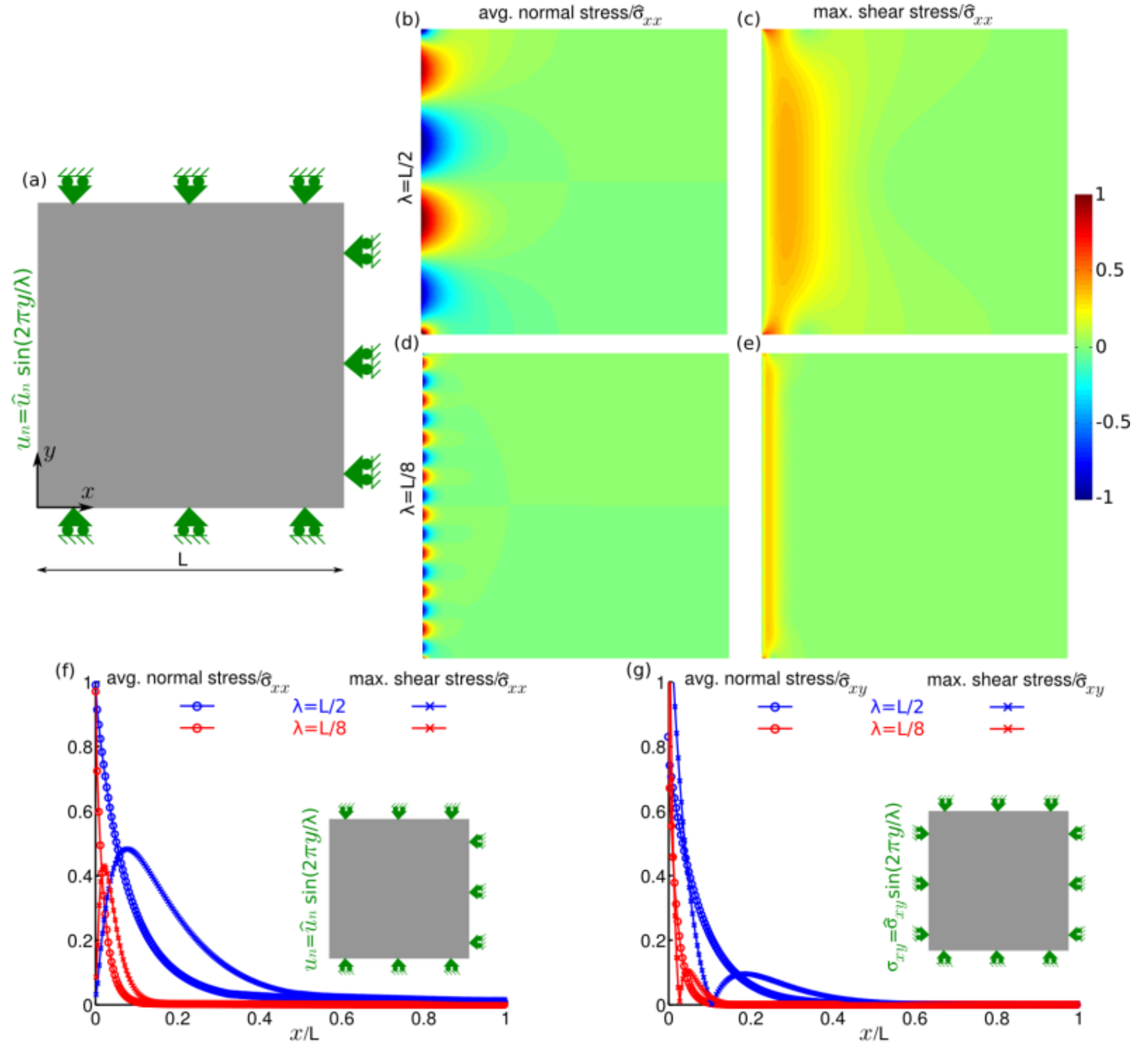

Figure S6. Propagation of boundary artifacts away from the optical edge for case 4. (a) A thin sheet subjected to sinusoidal perturbations in normal displacements $u_n = \hat{u}_n \sin(2\pi y/\lambda)$ at one edge, and $u_n = 0$ at two other edges. (b) Map of average normal stress, and (c) map of maximum shear stress when $\lambda = L/2$. (d-e) The stress maps when $\lambda = L/8$. (**f**) Decay of dominant Fourier mode in the stresses induced by the boundary conditions shown in the inset. Blue curves correspond to $\lambda = L/2$, and red curves correspond to $\lambda = L/8$. The curves marked with circle represent the induced average normal stress, and the curves marked with cross represent the induced maximum shear stress. (g) Decay curves of the stresses induced by boundary conditions shown in the inset. At all the boundaries along appropriate axis the natural boundary conditions, i.e. boundary stress=0 are not mentioned but they are implied. The stresses in (b-f) are normalized with the amplitude of induced normal stress $\hat{\sigma}_{xx}$ at the perturbed edge, the stresses in (g) are normalized with amplitude of imposed shear stress $\hat{\sigma}_{xy}$.

Experimental results for cases 2-4:

Artifacts attributable to the material properties:

For cases 2-4, we quantified the artifacts attributable to material properties by using the same approach as for case 1 (Fig. 4) and generated the associated scatter plots (Fig. S7).

Compared to the effect of heterogeneous elastic modulus, the effect of Poisson's ratio was quite small. Overall effect on case 2 and case 3 was similar, compared to which, the effect on case 4 was least.

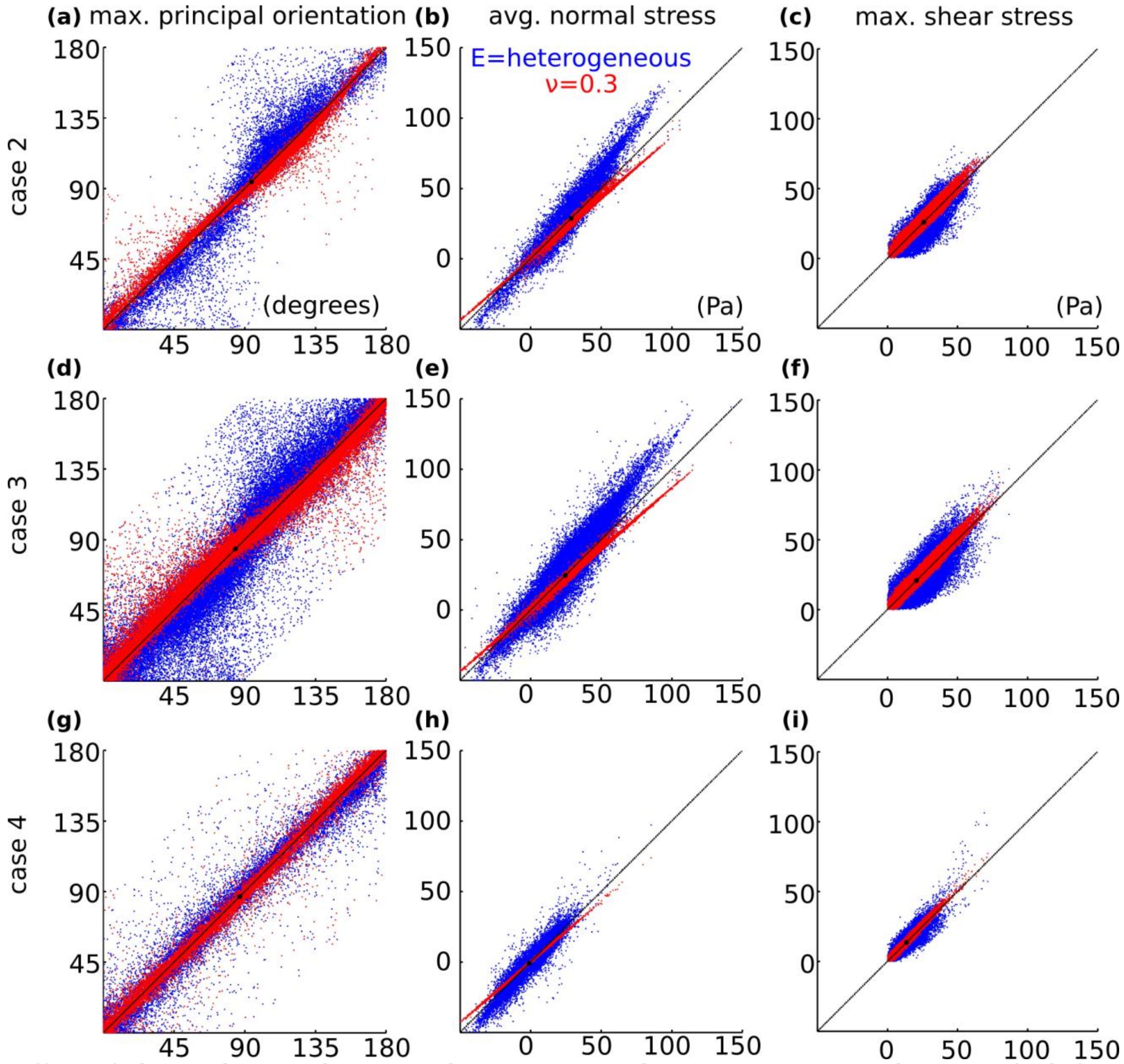

Figure S7. Effect of change in material properties on stresses in case 2, case 3, and case 4. (**a**,**d**,**g**) Scatter plots for maximum principal orientation where, red points quantify effect of ν, and blue points quantify effect of heterogeneity of E. (**b**,**e**,**h**) Scatter plots for average normal stress. (**c**,**f**,**i**) Scatter plots for maximum shear stress. Regression parameters for a straight line fit, $\delta y_i = m\ \delta x_i + c$, case 2: blue points, (f) $m = 1.1, c = 0.03\,°, r^2 = 0.9$, (g) $m = 1.24, c = 5.17$Pa$, r^2 = 0.94$, (h) $m = 0.87, c = -2.13$Pa$, r^2 = 0.64$; red points, (f) $m = 0.91, c = -0.02\,°, r^2 = 0.97$, (g) $m = 0.88, c = -3.64$Pa$, r^2 = 1$, (h) $m = 1.03, c = 2.11$Pa$, r^2 = 0.93$. Case 3: blue points, (f) $m = 1.04, c = -0.01\,°, r^2 = 0.89$, (g) $m = 1.21, c = 3.35$Pa$, r^2 = 0.91$, (h) $m = 0.88, c = -1.3$Pa$, r^2 = 0.69$; red points, (f) $m = 0.91, c = 0.01\,°, r^2 = 0.97$, (g) $m = 0.87, c = -3.19$Pa$, r^2 = 1$, (h) $m = 1.04, c = 1.95$Pa$, r^2 = 0.95$. Case 4: blue points, (f) $m = 0.95, c = -0.01\,°, r^2 = 0.95$, (g) $m = 1.1, c = -0.5$Pa$, r^2 = 0.9$, (h) $m = 0.93, c = -0.29$Pa$, r^2 = 0.75$; red points, (f) $m = 1, c = 0\,°, r^2 = 0.98$, (g) $m = 0.86, c = 0.13$Pa$, r^2 = 1$, (h) $m = 1.02, c = 0.85$Pa$, r^2 = 0.97$.

Supporting Information S5. Rate of decay of boundary artifacts: effect of adjacent free edges. At the optical edge, the normal displacements as well as the tangential tractions introduce artifacts that decay with increase in distance from the edge (Fig. 6). The decay of the artifacts from normal displacements is insensitive to the boundary conditions on other edges (Figs. 6f, and 6h). The decay of the artifacts from tangential tractions is, however, rapid when the adjacent edge is an optical edge

as opposed to a free edge (Fig. 6g vs. Fig. 6i; re-plotted on a log scale in Fig. S8). This sort of rapid decay of the boundary artifacts is the source of superior correlations between the stresses from case 4 and the stresses from gold standard.

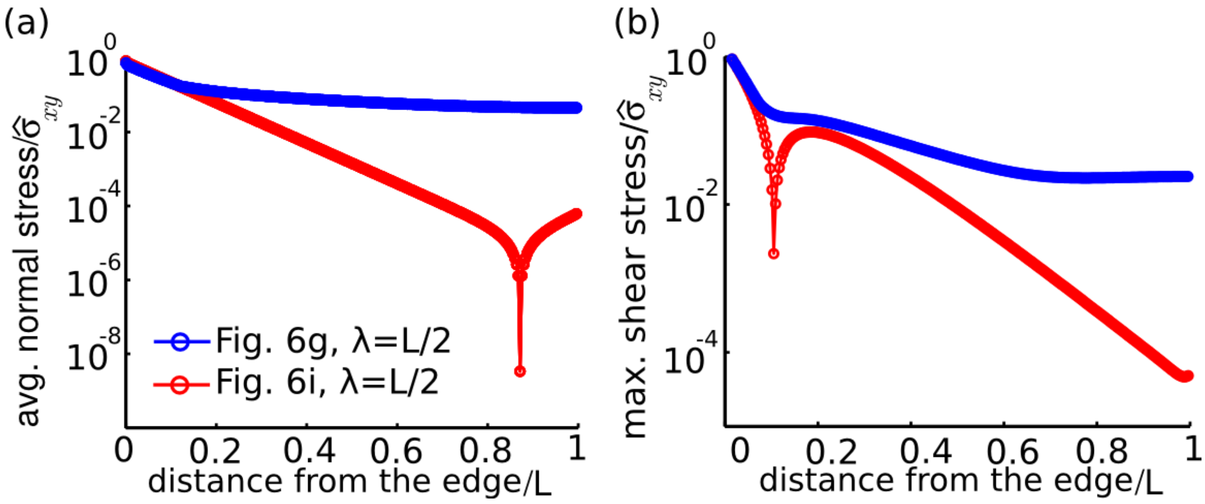

Figure S8. Rate of decay of the boundary artifacts of an edge is affected by boundary conditions at the adjacent edges. (a) Average normal stress, and (b) maximum shear stress, induced by shear stress perturbations of wavelength $\lambda = L/2$, are plotted as a function of distance from the perturbed edge. Away from the perturbed edge, the stresses decay. The rate of stress decay is, however, faster if the perturbed edge is opposite to the free edge (red curves) instead of being adjacent to the free edge (blue curves).

Supporting Information S6. Mapping the decay of boundary artifacts.
In our numerical analysis, we found that the greater the spatial frequency of errors at the boundary the faster is the decay of boundary artifacts (Figs. 6, S4, S6). This result was quantified by plotting the amplitude of dominant mode of a one dimensional Fourier transform (along the axis parallel to the perturbed edge) of the stresses as a function of distance from the boundary.

Supporting Information S7. Substrate tractions: effect of substrate thickness.
When the lateral scale of traction fluctuations (l) is greater than or equal to substrate thickness (H), then the effective stiffness sensed by the cells is larger than the actual stiffness of the substrate [8]. Below we examine whether the RPME cells in our monolayer are sensing substrate stiffness that is larger than actual.

In our experiments, we used substrates of thickness $H \approx 100\mu$m. From the spectral distribution of tractions, we found that the dominant contribution was from wavelengths close to 100μm (i.e. $l \approx H$, Fig. S9). However, compared to the tractions obtained from the algorithm of Butler et al. [4], the tractions obtained from the algorithm of Trepat et al. [3] were identical (Fig. S10). As such, the effect of substrate thickness on the distribution of tractions was minimal. Taken together, for RPME cell monolayers, when $H \approx 100\mu$m, the stiffness sensed by the cells approximates the actual stiffness of the substrate.

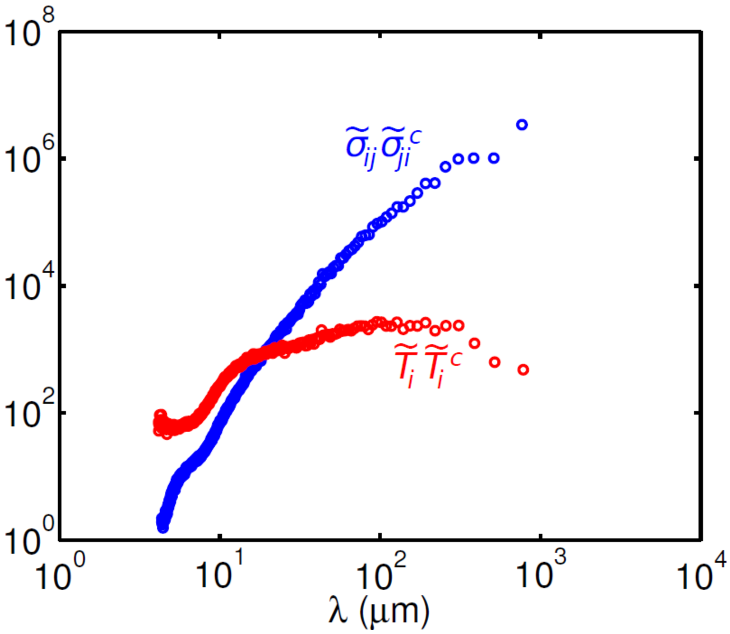

Figure S9. Spectral distribution of tractions and monolayer stresses. Red data points show squared magnitude of traction $(\tilde{T}_i)$, as a function of spectral wavelengths. Similarly, blue data points show the squared magnitude of monolayer stress $(\tilde{\sigma}_{ij})$. In the spectrum of the tractions, the wavelengths close to the value of thickness of the substrate (100 μm) contribute most. In contrast, in the spectrum of the monolayer stresses, all the wavelengths contribute in a manner that is sharply increasing with wavelength.

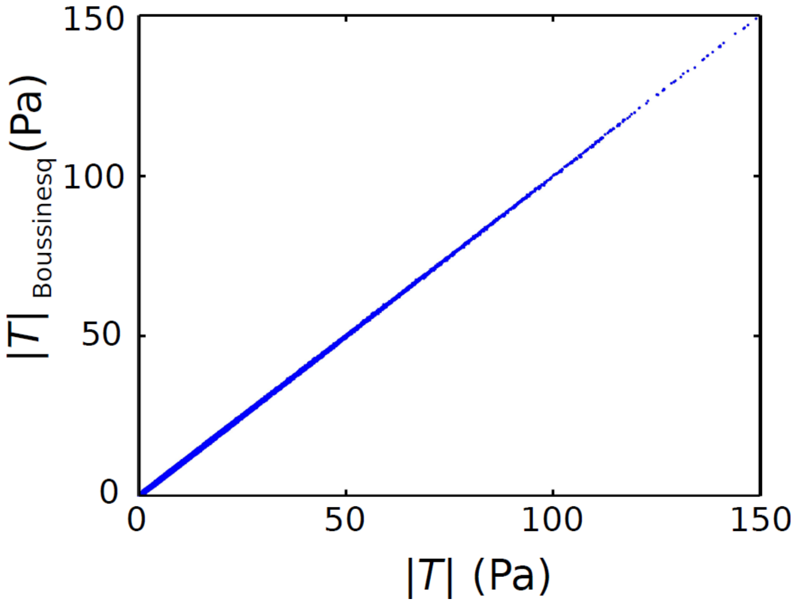

Figure S10. Tractions in Fig. 1 have negligible contribution from finite thickness of the substrate. Scatter plot of magnitude of tractions obtained from two-dimensional finite thickness algorithm [3] ($|T|$, substrate thickness = 100 μm) and two-dimensional Boussinesq algorithm [4] ($|T|_{Bousinesq}$). Through this scatter plot, regression fit of a straight line ($y = mx + c$) produces $m = 1.0, c = 0.01, r^2 = 0.99$.

Supporting Information S8. Enlarged image of selected results

Principal stress orientations shown in Fig. 4.

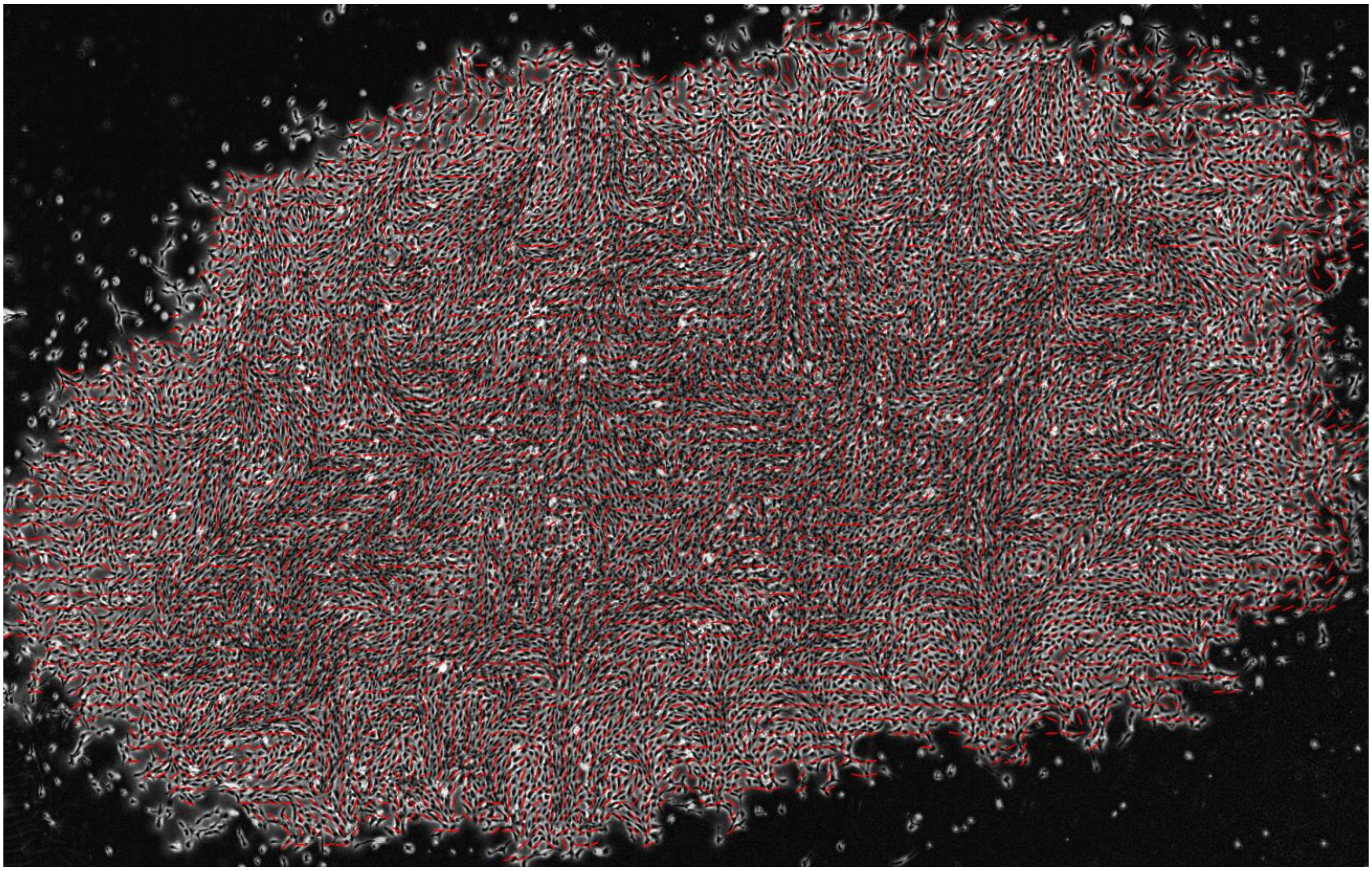

Figure S11. Enlarged version of Fig. 4a

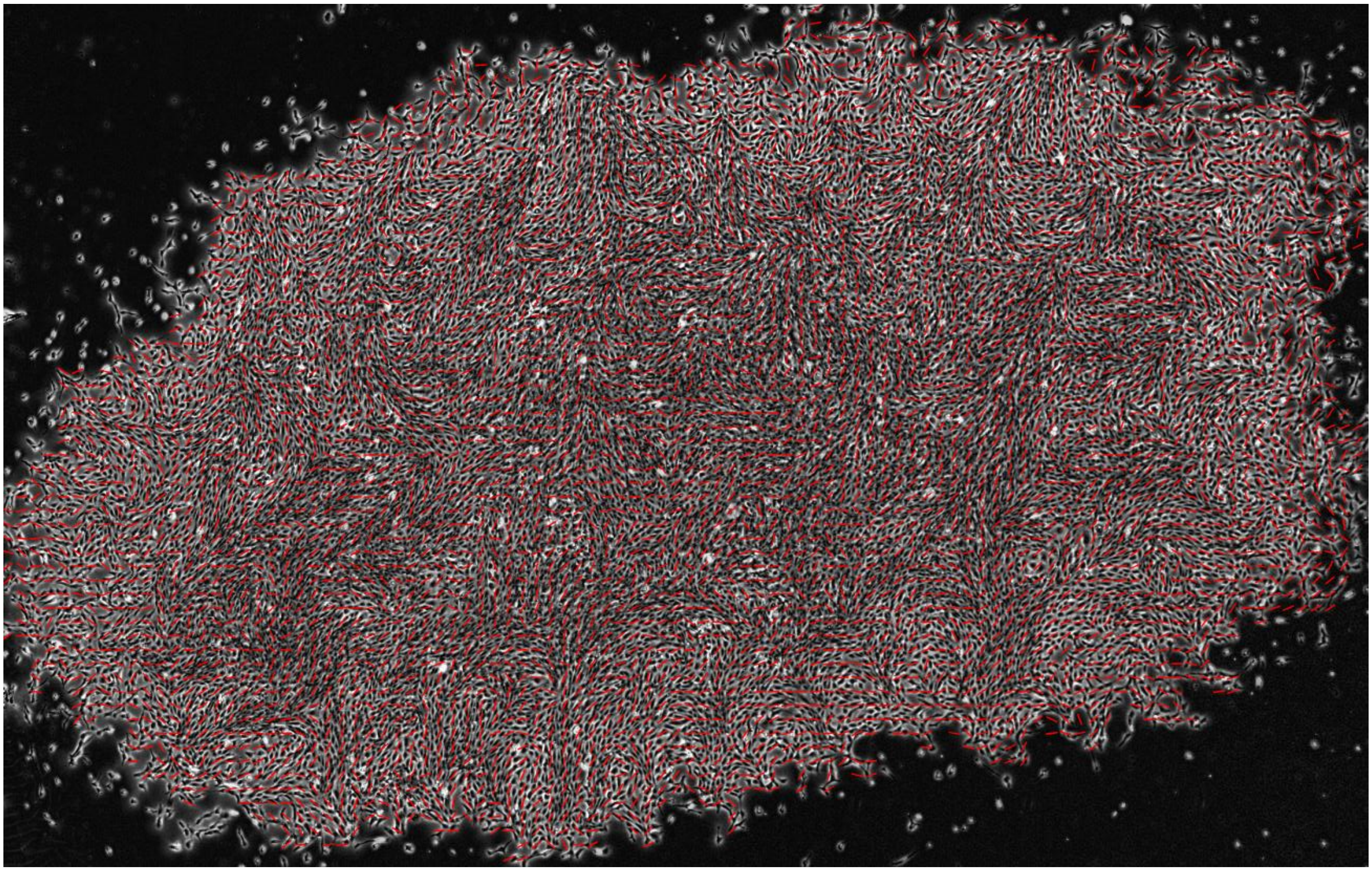

Figure S12. Enlarged version of Fig. 4d

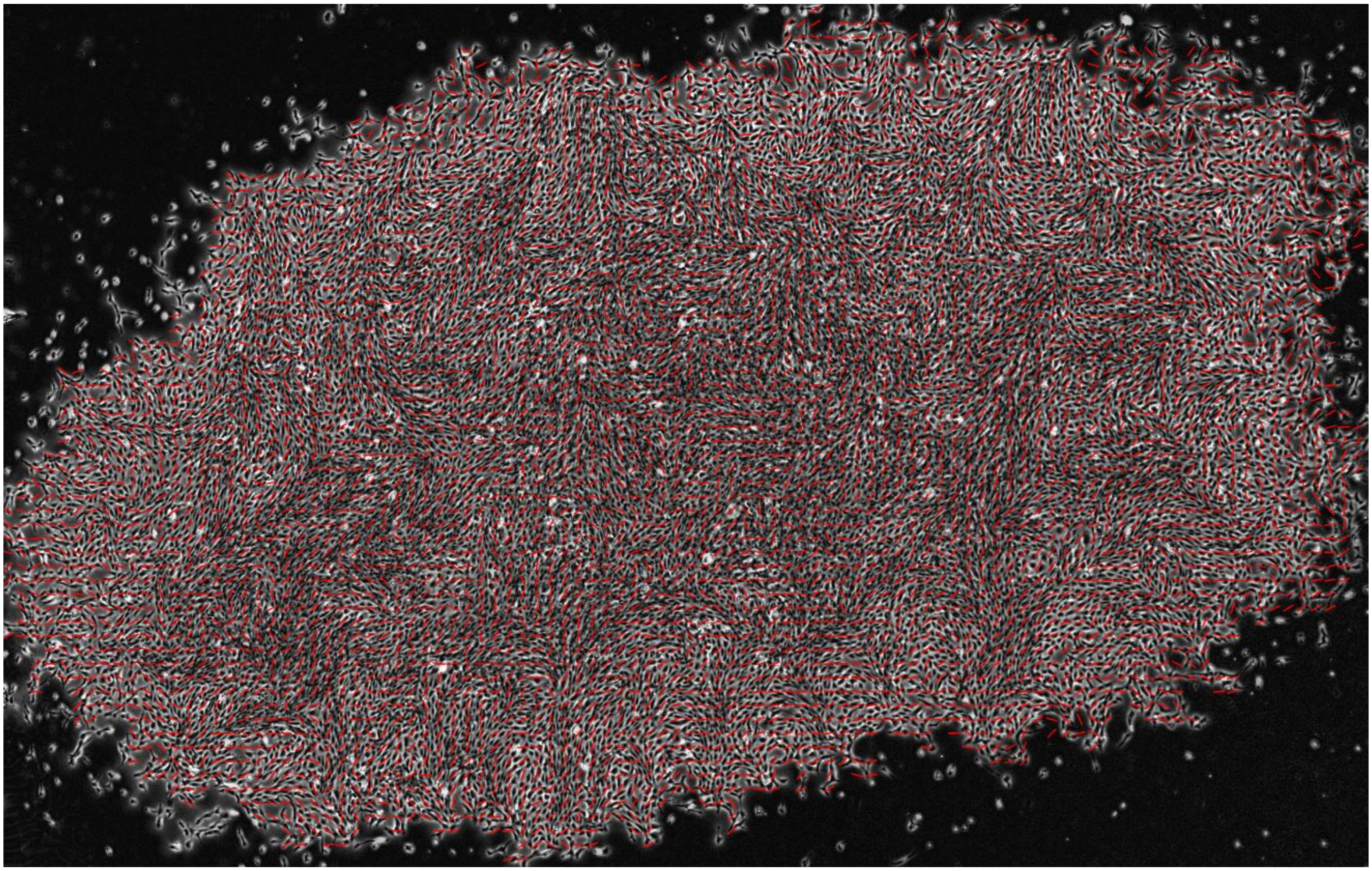

Figure S13. Enlarged version of Fig. 4g

Principal stress orientations shown in Fig. S3.

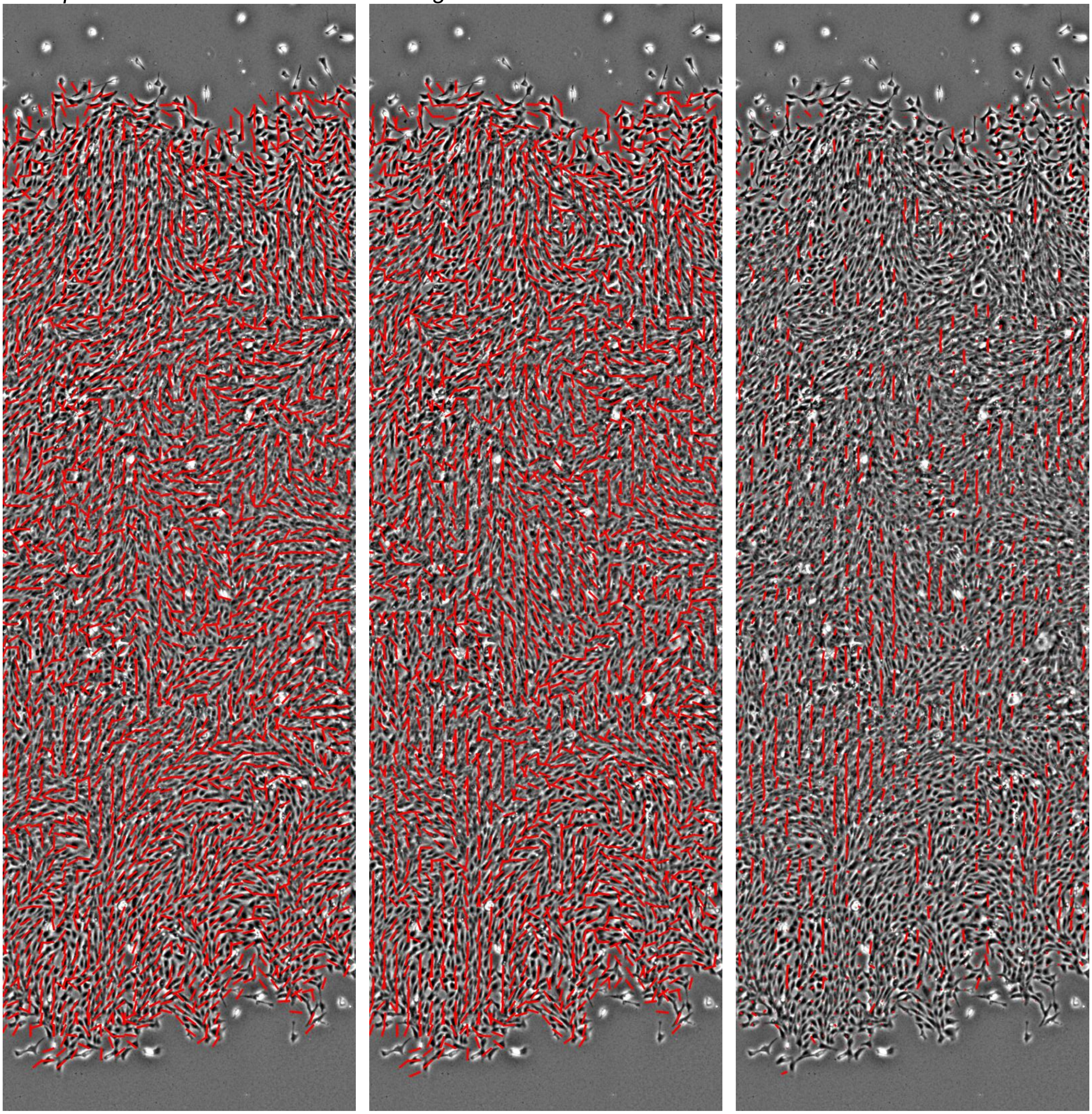

Figure S14. Enlarged version of Figs. S3a, S3d, S3g

References

1. An SS, Pennella CM, Gonnabathula A, Chen J, Wang N, et al. (2005) Hypoxia alters biophysical properties of endothelial cells via p38 MAPK- and Rho kinase-dependent pathways. American Journal of Physiology - Cell Physiology 289: C521-530.
2. Tambe DT, Hardin CC, Angelini TE, Rajendran K, Park CY, et al. (2011) Collective cell guidance by cooperative intercellular forces. Nat Mater 10: 469-475.
3. Trepat X, Wasserman MR, Angelini TE, Millet E, Weitz DA, et al. (2009) Physical forces during collective cell migration. Nat Phys 5: 426-430.
4. Butler JP, Tolic-Norrelykke IM, Fabry B, Fredberg JJ (2002) Traction fields, moments, and strain energy that cells exert on their surroundings. Am J Physiol Cell Physiol 282: C595-605.
5. Poujade M, Grasland-Mongrain E, Hertzog A, Jouanneau J, Chavrier P, et al. (2007) Collective migration of an epithelial monolayer in response to a model wound. Proceedings of the National Academy of Sciences 104: 15988-15993.
6. Serra-Picamal X, Conte V, Vincent R, Anon E, Tambe DT, et al. (2012) Mechanical waves during tissue expansion. Nature Physics In press.
7. Hur SS, del Alamo JC, Park JS, Li YS, Nguyen HA, et al. (2012) Roles of cell confluency and fluid shear in 3-dimensional intracellular forces in endothelial cells. Proc Natl Acad Sci U S A 109: 11110-11115.
8. Lin Y-C, Tambe DT, Park CY, Wasserman MR, Trepat X, et al. (2010) Mechanosensing of substrate thickness. Physical Review E 82: 041918.
